# Comparative Insights into Bacterial Endophytome and Antifungal Metabolites in *Fusarium* Wilt-Resistant and -Susceptible Banana Cultivars Unveil the Resistance Toward *Fusarium oxysporum* f. sp. *cubense*

**DOI:** 10.3390/pathogens15080864

**Published:** 2026-08-19

**Authors:** Dipti Pandurang Mohite, Kavino Mathiyazhagan, Nakkeeran Sevugapperumal, Raveendran Muthurajan, Raghu Rajasekaran, Saranya Nallusamy, Karunakaran Ganesan

**Affiliations:** 1 Department of Fruit Science, Horticultural College and Research Institute, Tamil Nadu Agricultural University, Coimbatore 641003, India; diptimohite2411@gmail.com; 2 Department of Plant Biotechnology, Centre for Plant Molecular Biology and Biotechnology, Tamil Nadu Agricultural University, Coimbatore 641003, India; nakkeeranayya@tnau.ac.in (N.S.); raghu.r@tnau.ac.in (R.R.); 3 Directorate of Research, Tamil Nadu Agricultural University, Coimbatore 641003, India; raveendrantnau@gmail.com; 4 Department of Plant Molecular Biology and Bioinformatics, Centre for Plant Molecular Biology and Biotechnology, Tamil Nadu Agricultural University, Coimbatore 641003, India; saranya.n@tnau.ac.in; 5 Division of Fruit Crops, ICAR-Indian Institute of Horticultural Research, Hesaraghatta, Bengaluru 560089, India; karanstg@gmail.com

**Keywords:** microbiome, *Fusarium*, endophytes, banana, metabolites

## Abstract

India contributes 26% of global banana production, yet cultivation is severely threatened by *Fusarium* wilt (*Fusarium oxysporum* f. sp. *cubense*, *Foc*), necessitating sustainable, eco-friendly management strategies. This study evaluated the biocontrol potential of endophytic bacteria isolated from *Foc*-resistant and -susceptible banana cultivars. Isolates from the pseudostem, corm, and root of the resistant cultivar showed significantly greater inhibitory activity than those from the susceptible cultivar, underscoring the role of host genotype in shaping functionally competent endophytic communities. Among all isolates, *Bacillus* sp. from the corm of cv. Rose (AB) showed the highest mycelial inhibition of *Foc* (70.37–76.54%) in vitro. GC-MS-based metabolite profiling revealed a chemically diverse array of secondary metabolites, fatty acid esters, steroids, terpenoids, siloxanes, and nitrogenous compounds. Corm-associated endophytes exhibited membrane-disruptive and cytotoxic activity, while root-associated endophytes contributed protective, defense-modulatory effects. *Halomonas* sp. RoRo2 and *B. subtilis* RoC1 showed the highest inhibition in both agar-well and in planta assays, with unsaturated fatty acids and organic acid derivatives implicated as key antifungal effectors acting through multiple biochemical pathways. These findings identify metabolically versatile endophytes as promising candidates for developing efficient, environmentally compatible bioinoculants as sustainable alternatives to chemical control of *Fusarium* wilt in banana.

## 1. Introduction

In India, banana (*Musa* spp.) is more than a staple fruit with a cultivation history deeply interwoven with the civilization [1]. Its historical and socioeconomic importance is also complemented by its modern agricultural dominance. India is one of the world’s largest producers of bananas, contributing a substantial 26% to the global production volume [2]. The 2023–2024 agricultural year underscored this prominence, with the crop cultivated across 0.94 million hectares, yielding an impressive 37.6 million tons and achieving a notable productivity rate of 40 tons per hectare [3]. This immense output highlights the crop’s critical role in ensuring rural livelihoods and national food security.

A primary threat to the global banana production is *Fusarium* wilt, a devastating vascular disease caused by the soil-borne, hemi-biotrophic ascomycete fungus *Fusarium oxysporum* f. sp. *cubense* (*Foc*). The pathogen’s infection cycle begins in the root system, often through minor wounds, after which it invades and systematically colonizes the vascular tissue of the rhizome. The plant’s defense mechanisms, while attempting to compartmentalize the invasion, contribute to its own demise. This includes the rapid formation of tyloses, outgrowths of parenchyma cells, and the secretion of gums and gels into the xylem vessels. This, combined with the accumulation of fungal biomass, leads to comprehensive vascular occlusion, while the oxidation of phenolic compounds produces the characteristic reddish-brown discoloration. This blockage severely impedes the translocation of water and nutrients, culminating in the classic symptoms of wilting, plant collapse, and ultimately, death [4]. Concurrently, the fungus secretes phytotoxins, such as fusaric acid, which disrupt physiological processes and induce chlorosis observed in the leaf lamina [5]. A critical challenge in managing this disease is the pathogen’s remarkable resilience. *Foc* produces chlamydospores that allow it to persist in a dormant state in the soil for decades, making crop rotation economically unviable and facilitating its spread through infected planting material, soil, and water [4].

Current management strategies for *Fusarium* wilt are limited and often impractical for banana growers. While the deployment of genetically resistant cultivars remains the most consistently recommended strategy, its widespread adoption is constrained, and development of these cultivars is a labor-intensive and costly affair. *Foc* resistant cultivar, particularly those derived from wild relatives, frequently possess traits like inferior fruit quality, lower yield, or smaller bunch size which are unacceptable to both growers and consumers [6]. This dilemma has necessitated a paradigm shift in plant pathology, moving beyond host genetics to explore the intricate role of the plant’s associated microbiome in conferring disease resistance.

A growing body of evidence now indicates that a plant’s resilience is not solely a function of its intrinsic genetics but is significantly mediated by the diverse community of microorganisms residing within its tissues, collectively known as the endomicrobiome [7]. Endophytic bacteria are recognized as essential components of the plant’s “second genome,” living symbiotically within the apoplast and vascular system without causing disease [8]. They contribute to host fitness through multiple mechanisms, broadly categorized as direct antagonism against pathogens and the induction of systemic resistance (ISR) [9]. The significance of this microbial shield is highlighted by comparative microbiome studies of *Foc*-resistant and susceptible banana genotypes. Resistant accessions, such as the wild diploid *Musa acuminata*, have been shown to harbor a more phylogenetically diverse and distinct endophytic community compared to susceptible commercial cultivars like Cavendish. The microbiome associated with resistant cultivars is often enriched with specific bacterial taxa from the Actinobacteria, γ-Proteobacteria, and Firmicutes phyla, which are frequently reported to possess potent bio control traits [10,11,12].

These studies highlight the biocontrol potential of a targeted screening strategy for identifying novel endophytic bacteria with antagonistic activity against *Foc*. Such a proactive strategy is essential for identifying strains capable of suppressing the pathogen through a combination of antibiosis, competition, parasitism, and the enhancement of the host’s physiological resilience via ISR [13]. The identification of such competent endophytes is the first, crucial step toward developing effective bio control formulations. Moreover, this foundational work enables the rational design of multi-strain microbial consortia. By strategically combining complementary endophytic strains with synergistic modes of action, it is possible to develop a more stable and effective biological product than traditional single-strain inoculants, which can fail under field conditions due to environmental variability and niche competition.

Therefore, this study was designed to systematically isolate, screen, and characterize endophytic bacteria from banana tissues for their efficacy in inhibiting *Foc* through in vitro assays. The ultimate objective is to identify potent native endophytes, which will serve as a resource for the future development of microbiome-based strategy to safeguard susceptible, commercially important cultivars and secure the long-term sustainability of global banana production against this devastating pathogen.

## 2. Materials and Methods

### 2.1. Isolation of Bacterial Endophytes

The bacterial endophytes from resistant and susceptible banana genotypes were isolated from root, corm, and pseudostem tissues from 11-month-old banana cultivars (Supplementary Figure S1). The resistant banana cultivar Rose (AB) and the susceptible cultivar Karpooravalli (ABB) were used for understanding the diversity of bacterial endophytome. These accessions were obtained from the germplasm collection maintained at the College Orchard (11°00′24″ N × 76°55′52″ E) of Department of Fruit Science, Horticultural College and Research Institute, Tamil Nadu Agricultural University (TNAU), Coimbatore-641003, Tamil Nadu, India. The banana germplasm was established under tropical climatic condition, and the soil parameters were as follows: pH ranged from 6.6 to 6.8, with the soil classified as sandy clay loam. The clay content was 15.70–15.90%, the silt content was 25.80–25.90%, and the sand content was 50.80–51.20%. Organic matter ranged between 1.45% and 1.74%, electrical conductivity (EC) varied from 0.19 to 0.23 dS m^−1^, and NPK content was 270:25:840 kg/ha. For each cultivar, samples were collected from 15 individual plants, and three representative samples were pooled before surface sterilization for the isolation of endophytic bacteria following the protocol of Bacon and Hinton [14].

The plant tissues were surface sterilized using 70% ethanol for 3 min, sodium hypochlorite (1%) for 3 min, and finally 4 rinses in sterile, distilled water. To confirm the effectiveness of the disinfection protocol, aliquots of the final rinse were plated onto Nutrient Agar plates as a sterility check and incubated at ambient temperature (28 ± 2 °C). Samples exhibiting microbial growth in the sterility checks within 48 h were discarded. Sterile samples were aseptically macerated in 10 mL of sterile potassium phosphate buffer (pH 7.0) using a sterile mortar and pestle. The homogenate was serially diluted up to 10^−5^ in the same buffer and 0.1 mL of each dilution was spread onto NA plates.

Plates were incubated at 28 ± 2 °C for 48 h in room condition. Bacterial colonies were selected based on distinct morphological characteristics such as colony shape, color, size, and growth pattern. Care was taken to avoid selecting morphologically identical colonies from the same plate to ensure diversity among isolates. Selected colonies were purified through repeated streaking on NA medium, and the purified single colonies were preserved in glycerol stocks at −80 °C for subsequent characterization and experimentation.

### 2.2. Dual Confrontation Assay

Dual confrontation assay provides an effective means to assess the antagonistic activity of one microorganism against another. In this study, we used dual culture assay to investigate the antagonistic interactions between endophytic microorganisms and *Foc* Race 1. The *Foc* Race1 pathogen was acquired from Department of Plant Pathology, TNAU. *Fusarium* culture was maintained on Potato Dextrose Agar (PDA) medium in sterile Petri plates.

The in vitro antifungal bioassays were carried out based on the dual culture method as previously described by Khamna et al. [15]. A 3 mm mycelium plug was excised from actively growing 5-day-old culture with a sterile cork borer and placed 1 cm away from the edge of sterile Petri plates containing PDA media. The bacterial endophyte was streaked on the opposite side of the fungal mycelium, 1 cm away from the edge of the same Petri plate. Plates containing the fungal plugs without bacterial inoculation were used as control plates. All plates were incubated at 28 ± 2 °C for five days. After incubation, the plates were examined for the evidence of antagonistic activity and inhibition zone. The reduction in mycelial growth over untreated control was calculated using the formula

$$\frac{C-T}{C}\times 100$$

where ‘C’ denotes the mycelial growth of the pathogen in control and ‘T’ denotes the mycelial growth of the pathogen in the dual-plate technique.

### 2.3. Extraction of Secondary Metabolites from the Antagonists

Volatile and non-volatile organic compounds (VOCs/NVOCs) produced by cultivable bacterial antagonists and diffused into the PDA medium from the zone of inhibition were carefully excised using a sterile scalpel. The agar sections containing VOCs/NVOCs were extracted by mixing 5 g of excised agar with 20 mL of HPLC grade acetonitrile in 1:4 ratio. The mixture was homogenized via sonication at 30% amplitude for 30 s, repeated twice to ensure thorough dispersion. Following homogenization, the samples were centrifuged to pellet residual solids and subsequently filtered to obtain a clear extract. The filtrate was concentrated by drying under reduced pressure using a vacuum flash evaporator (Roteva, Equitron Medica, Mumbai, India). The resulting dried extract was reconstituted in 1 mL of HPLC grade methanol for analysis. Differences in compound profiles were evaluated to determine specific VOCs/NVOCs induced or suppressed during pathogen–antagonist interaction.

### 2.4. Agar Well Diffusion Assay

The efficacy of extracted secondary metabolites from each bacterial antagonist was evaluated by the well diffusion assay. Four wells were made using cork borer at equal distance in the 9 cm petri dishes containing fresh sterilized PDA media (20 mL). 50 µL, 100 µL, and 200 µL concentrations of the extracted secondary metabolite of each antagonist were poured in each of the wells separately, and the mycelial disc of each pathogen was placed in the center of the plates separately. The wells filled with methanol alone and the mycelial disc of each pathogen placed at the center of each petri plate served as control. The mycelial growth of the pathogen was recorded, and the percent of reduction in the mycelial growth over control was calculated [16].

### 2.5. Molecular Characterization

Genomic DNA from bacterial isolates was extracted following the method of Green and Sambrook [17]. Overnight-grown cultures were lysed using SDS, lysozyme, and RNase, followed by purification with chloroform, as follows: isoamyl alcohol and precipitation using sodium acetate and isopropanol. The DNA pellet was washed with 70% ethanol, air dried, and dissolved in TE buffer (10 mM Tris-HCl, 1 mM EDTA, pH 8.0). DNA quality was verified on 1% agarose gel electrophoresis stained with ethidium bromide, and concentration was determined using a Nanodrop Spectrophotometer (Genova Nano, Genway, San Diego, CA, USA).

PCR amplification of the bacterial 16S rRNA gene was carried out using universal primers 27F (5′-AGAGTTTGATCCTGGCTCAG-3′) and 1492R (5′-ACGGCTACCTTGTTACGACTT-3′) [18] sourced from Sigma-Aldrich (Merck Life Science) Bangalore, India. The reaction mixture (25 µL) is presented in Table 1. The amplification program consists of an initial denaturation at 95 °C for 3 min, followed by 35 cycles of 95 °C for 30 s, 58 °C for 30 s, and 72 °C for 90 s, with a final extension at 72 °C for 5 min. PCR products were analyzed on a 1% agarose gel and further sequenced. Sanger sequences generated in this study were submitted to the NCBI database, and a neighbor joining phylogenetic tree was constructed using MEGA software version 12.

### 2.6. Pot Experiment

A pot culture experiment was conducted to assess the in vivo biocontrol efficacy of 12 culturable bacterial endophytes against *Fusarium* wilt of banana. The experiment was carried out at the Horticultural College and Research Institute (HC & RI), Tamil Nadu Agricultural University (TNAU), Coimbatore, India, and was laid out in a Completely Randomized Design (CRD) with three replications per treatment, each replication comprising ten plants. Polyhouse conditions were maintained at 29–32 °C and 70% relative humidity, under natural sunlight with a cladding material providing 90% light transmission, with an average photosynthetically active radiation (PAR) of 800–1000 µmol/m^2^/s, under a natural photoperiod of 11.5 h light and 12.5 h dark throughout the experimental period. Cocopeat was used as the potting medium; prior to use it was rinsed three times with water and sterilized by autoclaving at 121 °C for 50 min to eliminate microbial contamination. Uniform, primary hardened-tissue cultured plantlets of banana cv. Karpoorvalli were selected for the study.

For inoculum preparation, each bacterial isolate was grown in Nutrient Broth (NB) at 28 °C for 48 h under continuous shaking, and the resulting suspension was standardized to a cell density of 1 × 10^8^ CFU mL^−1^. The pathogen inoculum was prepared separately, with the conidial suspension adjusted to 10^5^ spores mL^−1^ before inoculation.

The treatments were grouped into three categories, as follows: an uninoculated control (healthy plants without any inoculation), a pathogen-alone treatment (plants challenged with *Foc* only), and combined inoculation (plants treated with an individual bacterial isolate together with *Foc*). At transplanting, the plants were biohardened by dipping the roots in the respective bacterial suspensions to ensure uniform bacterial colonization. Bacterial inoculation was subsequently repeated four times at 15-day intervals to ensure endophytic colonization within the host plant. The *Foc* pathogen was introduced on the 60th day after transplanting.

At the close of the experiment (90th day), a comprehensive set of morphological and growth parameters was recorded, namely root length, projected root area, root surface area, root diameter, root volume, root fresh weight, pseudostem height, pseudostem girth, number of leaves, leaf area, and phyllochron. The entire experiment was conducted in triplicate to confirm the reproducibility of the results. (Photographs of pot experiment treatments were taken using a Nikon D3400 digital SLR camera (Nikon Corporation, Ayutthaya, Indonesia) fitted with a 22 mm focal length lens (35 mm equivalent: 33 mm). All images were captured at an aperture of f/3.8, shutter speed of 1/125 s, and ISO-800, with no flash and 0 EV exposure bias. Images were captured against a uniform black background under consistent ambient lighting conditions to standardize visual comparison among treatments.)

#### Disease Index (DI)

*Foc* disease severity (Table 2) was assessed using the rating scale of Dita et al. [19]:

The Disease Index (%) was determined for each treatment using the following formula:

$$\mathrm{DI}\;(\%)\;=\;\sum \;\frac{\left(n\times v\right)}{\left(N\times V\right)}\times 100$$

where *n* is the number of plants in a given disease grade, *v* is the grade value (1–5), *N* is the total number of plants assessed per replication (10), and *V* is the maximum grade value (5). Three replications were evaluated for each treatment.

### 2.7. GC-MS Analysis

The secondary metabolites extracted from the zone of inhibition of *Foc* and bacterial antagonist were subjected to GC-MS analysis in Perkin Elmer Clarus SQ8C, Waltham, MA, USA, instrument at Department of Biotechnology, TNAU, Coimbatore, Tamil Nadu, India to identify the compound associated with the inhibition of the pathogen. Samples subjected to GC-MS analysis were extracted from *B. subtilis* RoC1, *B. inaquosorum* RoCo3, and *Halomonas* sp. RoRo2. These three isolates were selected from 12 culturable bacterial endophytes because they consistently ranked as the top performers across both the dual confrontation and agar well diffusion assays. Profiling was conducted to identify metabolites produced by the bacterial isolates under two conditions, as follows: (i) bacterial cultures grown individually, and (ii) bacterial cultures confronted with *Foc*. The obtained metabolite profiles were further compared with those produced by *Foc* alone, to elucidate the unique secondary metabolites synthesized during the interaction of bacteria and *Foc*.

The samples were loaded in DB-5 MS capillary standard nonpolar column 1 µL. The temperature was set at 30 °C for one minute and 230 °C at 4 °C/minute for 20 min. The sensor and the injector were heated at 250 °C and 230 °C, respectively. Helium gas was used as a carrier gas 5 PSI pressure. By ionizing the electrons at 70 eV, mass spectra were acquired. Using comparisons like retention time, retention indices, and mass spectra, the compounds were identified in National Institute of Standards and Technology’s spectrum data obtained from the NIST library (NIST Version 2.0, 2005) the GC-MS apparatus [20].

### 2.8. Statistical Analysis

The experiments were laid out in a Completely Randomized Design (CRD) and analysed in R (v4.5.1). For the pot study, treatment effects were tested by one-way ANOVA (*p* < 0.001), and mean separation was performed using Duncan’s Multiple Range Test (α = 0.05). Comparative metabolite interaction networks were constructed in Cytoscape (v3.10.3) to enable comprehensive visualization of the metabolites.

## 3. Results

**Figure 1:**
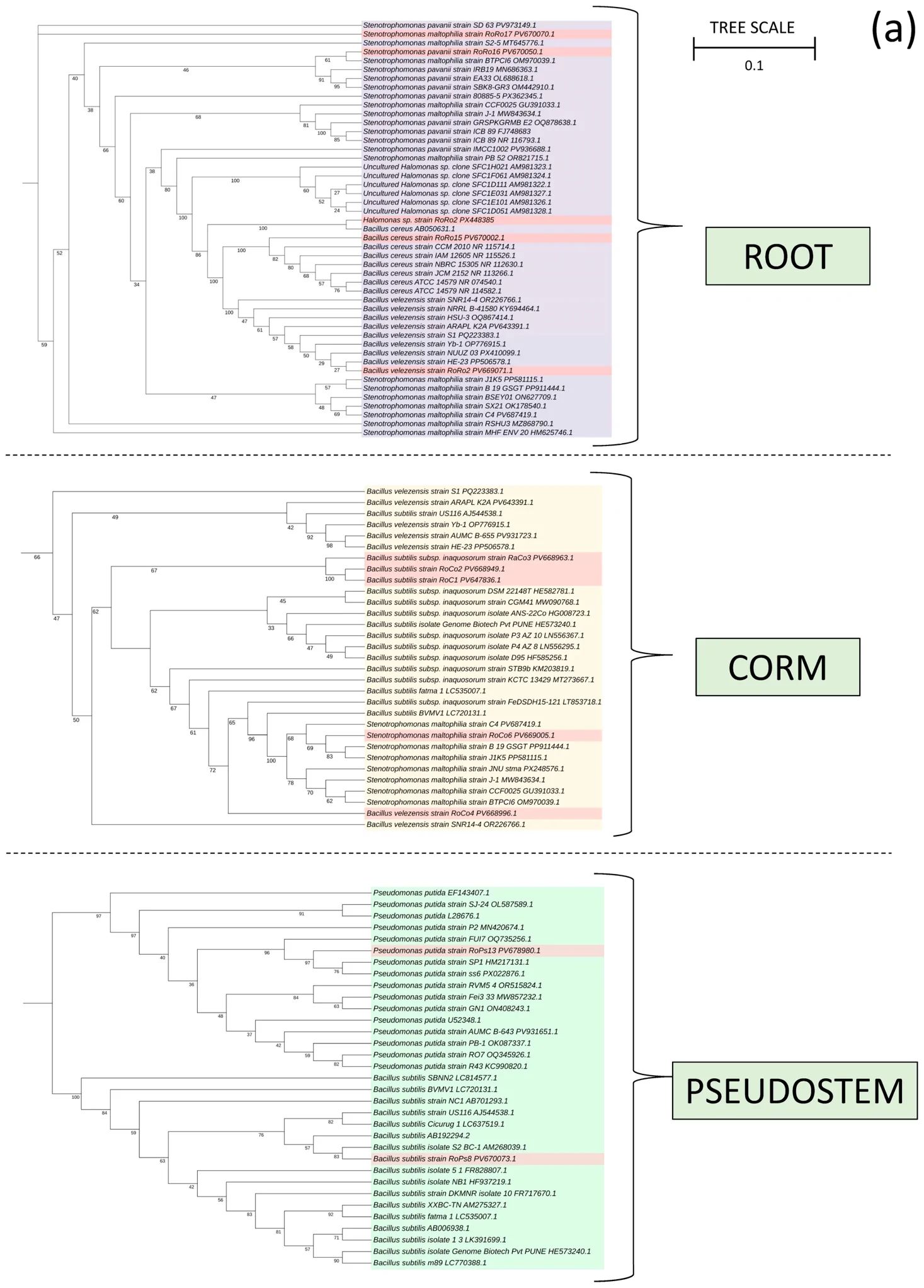

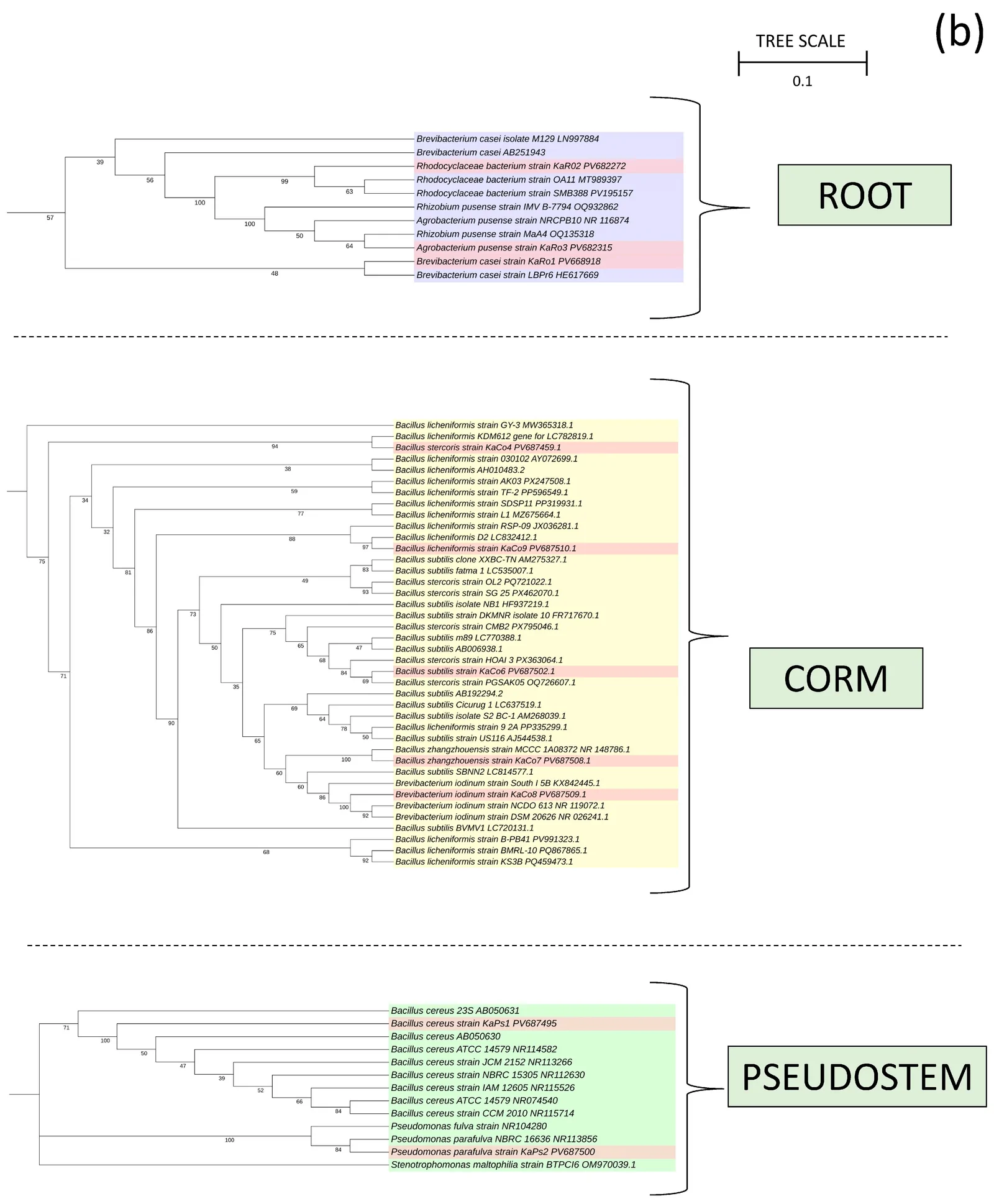
(**a**) Phylogenetic tree of endophytic bacterial strains isolated from resistant cultivar Rose (AB) and (**b**) susceptible cultivar Karpoorvalli (ABB). The strains highlighted in red are isolates used in this study.

The resistant banana cultivar Rose (AB) harbored a diverse array of endophytic bacterial isolates belonging to the genera *Bacillus*, *Stenotrophomonas*, *Halomonas*, *Pseudomonas,* and *Brevibacterium*. A highly diverse and rich endophytic microbiome was observed in the resistant cultivar compared to the susceptible cultivar *viz.*, Karpooravalli (ABB). A total of 42 culturable bacterial endophytes were successfully isolated from pseudostem, corm, and root of resistant banana cultivar, reflecting their rich microbial diversity, whereas only 12 culturable bacterial endophytes were obtained from susceptible cultivar. Among the isolates from resistant cultivar, 12 had more than 50% inhibition against *Foc* Race 1. The bacterial endophytes were subsequently identified by Sanger sequencing and deposited in NCBI GenBank database. The identified bacterial endophytes *Bacillus subtilis* strain RoC1 (PV647836), *Bacillus subtilis* strain RoCo2 (PV668949), *Bacillus inaquosorum* strain RoCo3 (PV668963), *Bacillus velezensis* strain RoCo4 (PV668996), and *Stenotrophomonas maltophilia* strain RoCo6 (PV669005) were isolated from corm. *Halomonas* sp. strain RoRo2 (PX448385), *Bacillus velezensis* strain RoRo2 (PV670015), *Bacillus cereus* strain RoRo15 (PV670002), *Stenotrophomonas pavanii* strain RoRo16 (PV670050), and *Stenotrophomonas maltophilia* strain RoRo17 (PV670070) were isolated from root. *Bacillus subtilis* strain RoPs8 (PV670073) and *Pseudomonas putida* strain RoPs13 (PV678980) were isolated from pseudostem in resistant cultivar (Table 3). The endophytes isolated from the root, corm, and pseudostem of the resistant cultivar were compared with reference sequences deposited in the NCBI database, and a neighbor- joining phylogenetic tree was constructed (Figure 1).

Similarly, all isolates obtained from the susceptible cultivar were identified as *Brevibacterium casei* strain KaRo1 (PV668918). *Rhodocyclaceae bacterium* strain KaRo2 (PV682272) and *Rhizobium pusense* strain KaRo3 (PV682315) were isolated from root; *Bacillus subtilis* subsp. *stercoris* strain KaCo4 (PV687459), *Bacillus subtilis* strain KaCo6 (PV687502), *Bacillus zhangzhouensis* strain KaCo7 (PV687508), *Brevibacterium iodinum* strain KaCo8 (PV687509), and *Bacillus licheniformis* strain KaCo9 (PV687510) were isolated from corm; and *Bacillus cereus* strain KaPs1 (PV687495) and *Pseudomonas parafulva* strain KaPs2 (PV687500) were isolated from pseudostem and submitted in NCBI database. The inhibition percentage varied significantly among the resistant and susceptible cultivars, demonstrating their differential antagonistic potential against the pathogen.

### 3.1. Dual Confrontation Assay

**Figure 2:**
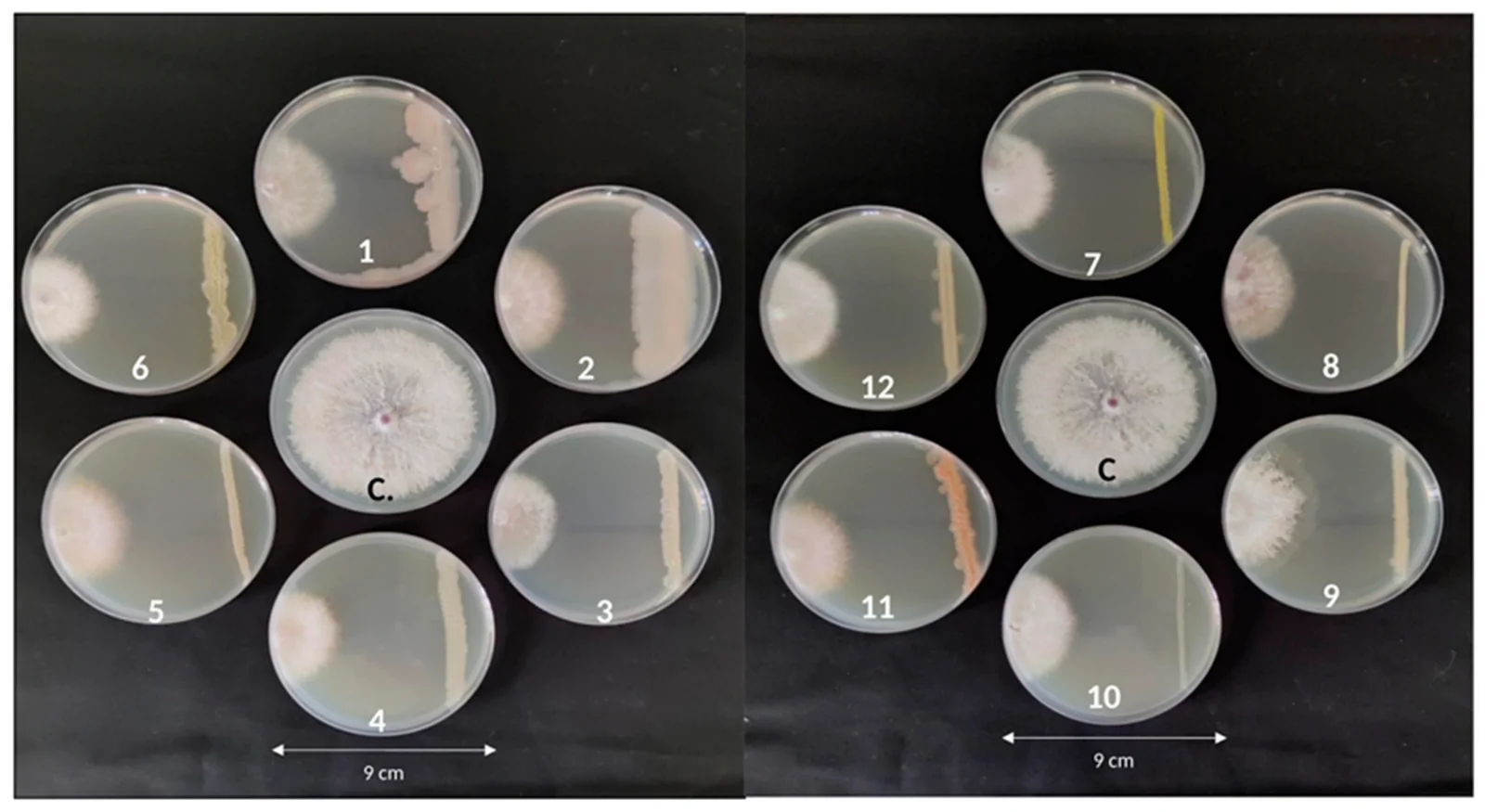
Dual confrontation assay of endophytic bacteria isolated from resistant cultivar Rose (AB). 1. *Bacillus subtilis* strain RoC1, 2. *Bacillus subtilis* strain RoCo2, 3. *Bacillus inaquosorum* strain RoCo3, 4. *Bacillus velezensis* strain RoCo4, 5. *Stenotrophomonas maltophilia* strain RoCo6, 6. *Halomonas* sp. strain RoRo2. 7. *Pseudomonas putida* strain RoPs13, 8. *Bacillus velezensis* strain RoRo2, 9. *Bacillus cereus* strain RoRo15, 10. *Stenotrophomonas pavanii* strain RoRo16, 11. *Bacillus subtilis* strain RoPs8, 12. *Stenotrophomonas maltophilia* strain RoRo17. C. Control. The same control plate image (C) was used across all treatments to enable a consistent reference for a fair visual comparison.

The antagonistic interaction between endophytic bacterial isolates and *Foc* was assessed through dual confrontation assay. The endophytes isolated from resistant banana cultivar demonstrated pronounced inhibitory effects on *Foc*, *B. subtilis* RoC1 (70.37%) which had the highest mycelial growth inhibition, followed by *Halomonas* sp. RoRo2 (69.55%). Comparable inhibition was observed for *S. maltophilia* RoRo17 (69.45%), *B. subtilis* RoCo2 (69.14%), and *S. pavanii* RoRo16 (69.14%). Further, comparative analysis of the inhibitory effect by the bacterial endophytes revealed that moderate antagonistic activity was observed in the endophytes including *Pseudomonas putida* RoPs13 (68.54%), *B. subtilis* RoPs8 (67.49%), *Halomonas* RoRo2 (66.26%), *B. inaquosorum* RoCo3 (65.84%), *B. velezensis* RoCo4 (64.61%) and *S. maltophilia* RoCo6 (63.37%). This assay clearly indicated that *B. subtilis RoC1* (70.37%) and *Halomonas* sp. RoRo2 (69.55%) possessed the most pronounced antifungal activity against *Foc* (Figure 2).

**Figure 3:**
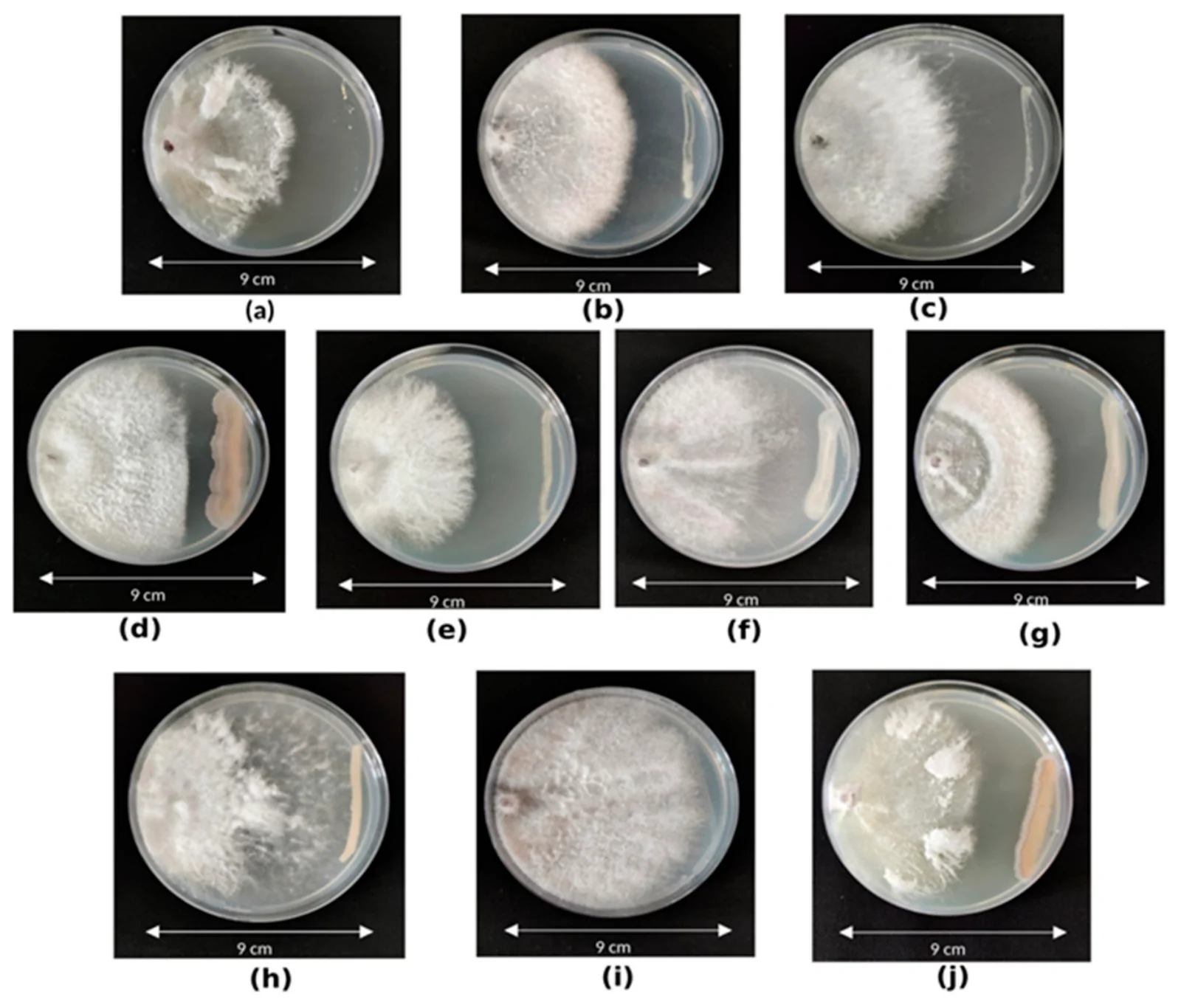
Dual confrontation assay of endophytic bacteria isolated from susceptible cultivar Karpooravalli (ABB) (**a**) *Brevibacterium casei* strain KaRo1 (PV668918); (**b**) *Rhodocyclaceae bacterium* strain KaRo2 (PV682272); (**c**) *Rhizobium pusense* strain KaRo3 (PV682315); (**d**) *Bacillus subtilis* subsp. *stercoris* strain KaCo4 (PV687459); (**e**) *Bacillus cereus* strain KaPs1 (PV687495); (**f**) *Pseudomonas parafulva* strain KaPs2 (PV687500); (**g**) *Bacillus subtilis* strain KaCo6 (PV687502); (**h**) *Bacillus zhangzhouensis* strain KaCo7 (PV687508); (**i**) *Brevibacterium iodinum* strain KaCo8 (PV687509); (**j**) *Bacillus licheniformis* strain KaCo9 (PV687510).

**Figure 4:**
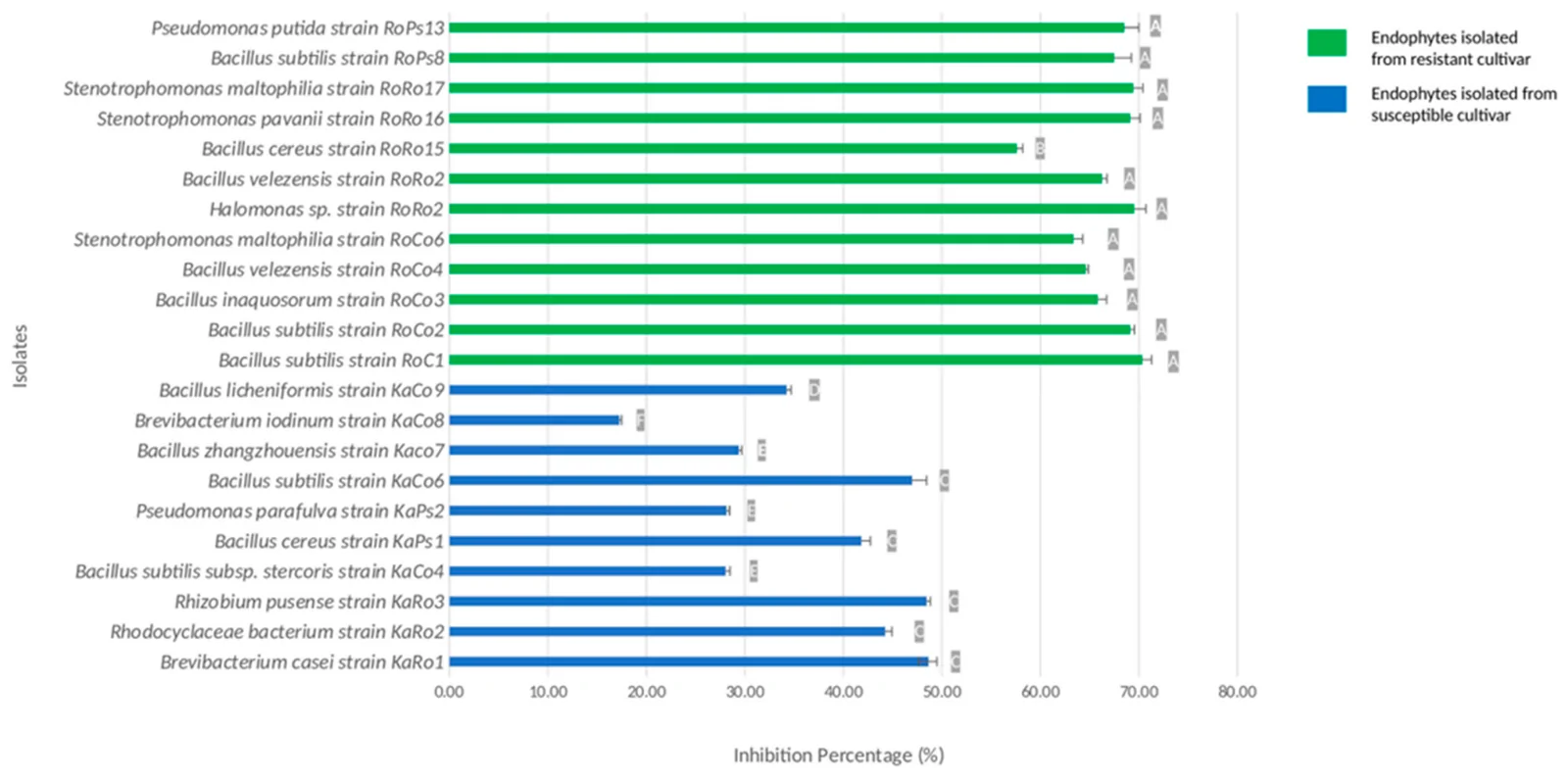
Antagonistic activity of endophytic bacterial strains isolated from *Fusarium oxysporum* f. sp. *cubense* resistant and susceptible cultivars. The uppercase letters denote statistically significant differences (Duncan’s Multiple Range Test (α = 0.05)).

Endophytic bacterial isolates from the susceptible banana cultivar also exhibited the antagonistic potential. Among isolates from susceptible cultivar, it ranged between 12.78% and 48.96% against *Foc*. *B. casei* KaRo1 (48.67); the latter and *R. pusense* KaRo3 (48.48) exhibited the highest values, followed closely by *B. subtilis* KaCo6 (46.99) and *R. bacterium* KaRo2 (44.26). Inhibition was recorded for *B. cereus* KaPs1 (41.84) and *B. licheniformis* KaCo9 (34.26). In contrast, lower values were observed for *B. subtilis* subsp. *stercoris* KaCo4 (28.05), *P. parafulva* KaPs2 (28.15), and *B. zhangzhouensis* KaCo7 (29.4), and the lowest for *B. iodinum* KaCo8 (17.27) (Figure 3). These results clearly indicate a comparatively lower antagonistic potential against *Foc* than endophytes isolated from resistant cultivar (Figure 4).

### 3.2. Agar Well Assay

**Figure 5:**
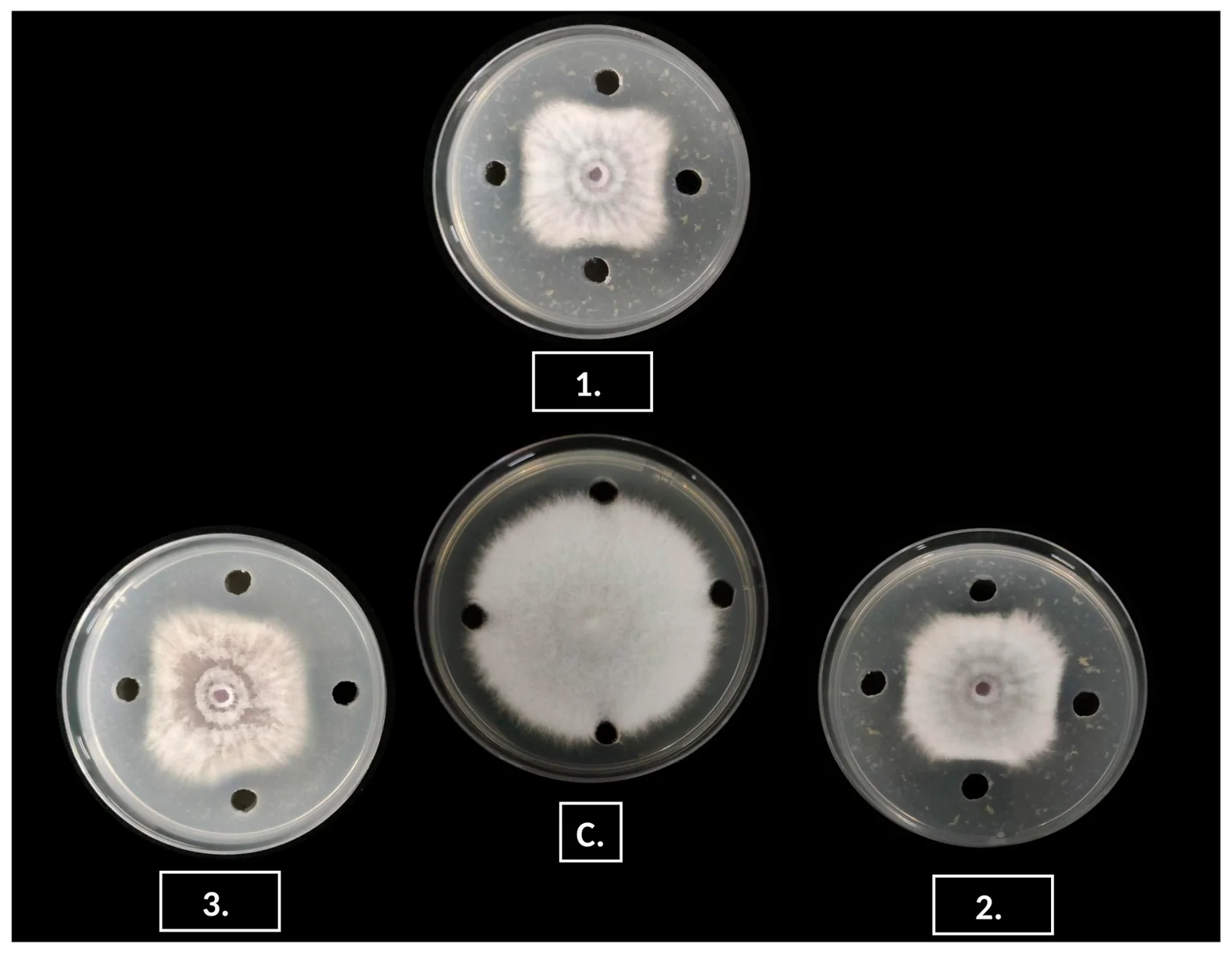
Agar well assay using endophytic bacteria isolated from resistant cultivar Rose (AB). 1. *Bacillus subtilis* strain RoC1 (PV647836), 2. *Bacillus inaquosorum* strain RoCo3 (PV668963), and 3. *Halomonas* sp. strain RoRo2 (PX448385), C. Control.

The secondary metabolites produced by bacterial isolates belonging to the genera *Bacillus*, *Stenotrophomonas*, and *Pseudomonas* exhibited significantly varying antagonistic activity. Isolates *B. inaquosorum* RoCo3 (76.24%), *Halomonas* sp. RoRo2 (75.81%), and *B. subtilis* RoC1 (74.06%) being the most promising strains (Figure 5). *B. subtilis* RoCo2 (66.99%) and *S. maltophilia* RoCo6 (68.22%) also showed antagonistic potential, though comparatively lower. The lowest inhibition was recorded for *B. subtilis* RoPs8 (36.86%), *B. cereus* RoRo15 (20.22%), *B. velezensis* RoCo4 (19.25%), *P. putida* RoPs13 (19.22%), and *S. pavanii* RoRo16 (17.25%). Overall, the metabolites produced by *Bacillus* and *Halomonas* species exhibited the highest antagonistic activity against *Foc*.

**Figure 6:**
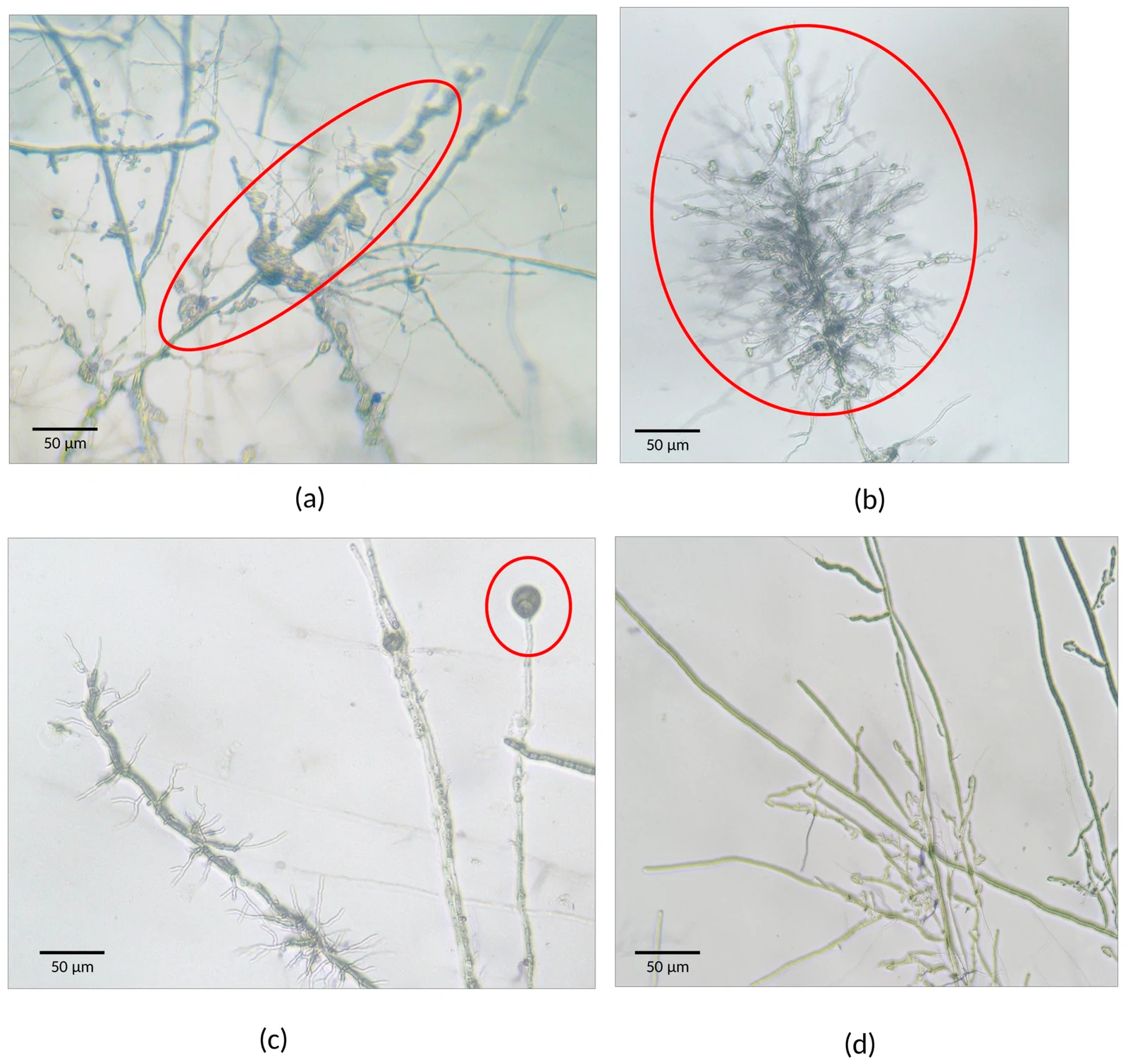
Bulging, curling, and irregular mycelial distortions observation (@ 100× magnification) in the interaction zone in the agar well assay, (**a**) Irregular mycelial in *Bacillus subtilis* strain RoC1 (PV647836); (**b**) Irregular mycelial and restricted growth in *Halomonas* sp. strain RoRo2 (PX448385); (**c**) Bulging in *Bacillus subtilis* subsp. *inaquosorum* strain RoCo3 (PV668963); (**d**) Control.

Microscopic examination of *Foc* hyphae within the interaction zone of the agar-well assays revealed that exposure to antifungal metabolites from *B. subtilis* RoC1, *B. inaquosorum* RoCo3, and *Halomonas* sp. RoRo2 induced distinct morphological alterations. The fungal hyphae exhibited evident structural deformities, including bulging, curling, and irregular mycelial distortions, suggesting severe disruption of cellular integrity (Figure 6). These morphological abnormalities provide clear evidence of the strong antagonistic effect exerted by these bacterial antifungal metabolites on *Foc*.

### 3.3. Pot Experiment

**Figure 7:**
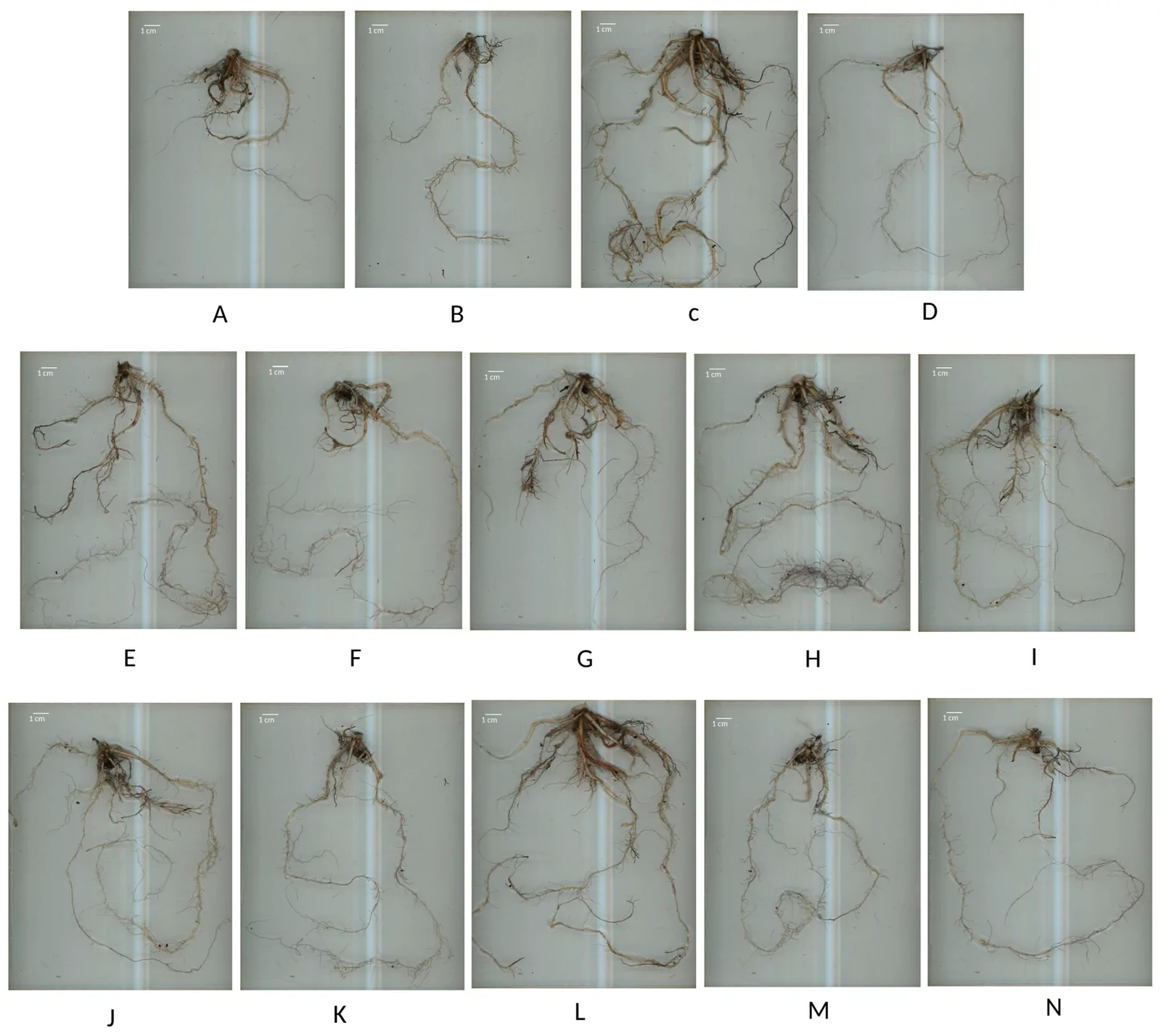
Root morphology of plants treated with endophytic bacteria isolated from resistant cultivar (**A**) Positive Control; (**B**) Negative Control; (**C**) *Bacillus subtilis* strain RoC1; (**D**) *Bacillus subtilis* strain RoCo2; (**E**) *Bacillus inaquosorum* strain RoCo3; (**F**) *Bacillus velezensis* strain RoCo4; (**G**) *Stenotrophomonas maltophilia* strain RoCo6; (**H**) *Halomonas* sp. strain RoRo2; (**I**) *Bacillus velezensis* strain RoRo2; (**J**) *Bacillus cereus* strain RoRo15; (**K**) *Stenotrophomonas pavanii* strain RoRo16; (**L**) *Stenotrophomonas maltophilia* strain RoRo17; (**M**) *Bacillus subtilis* strain RoPs8; (**N**) *Pseudomonas putida* strain RoPs13.

**Figure 8:**
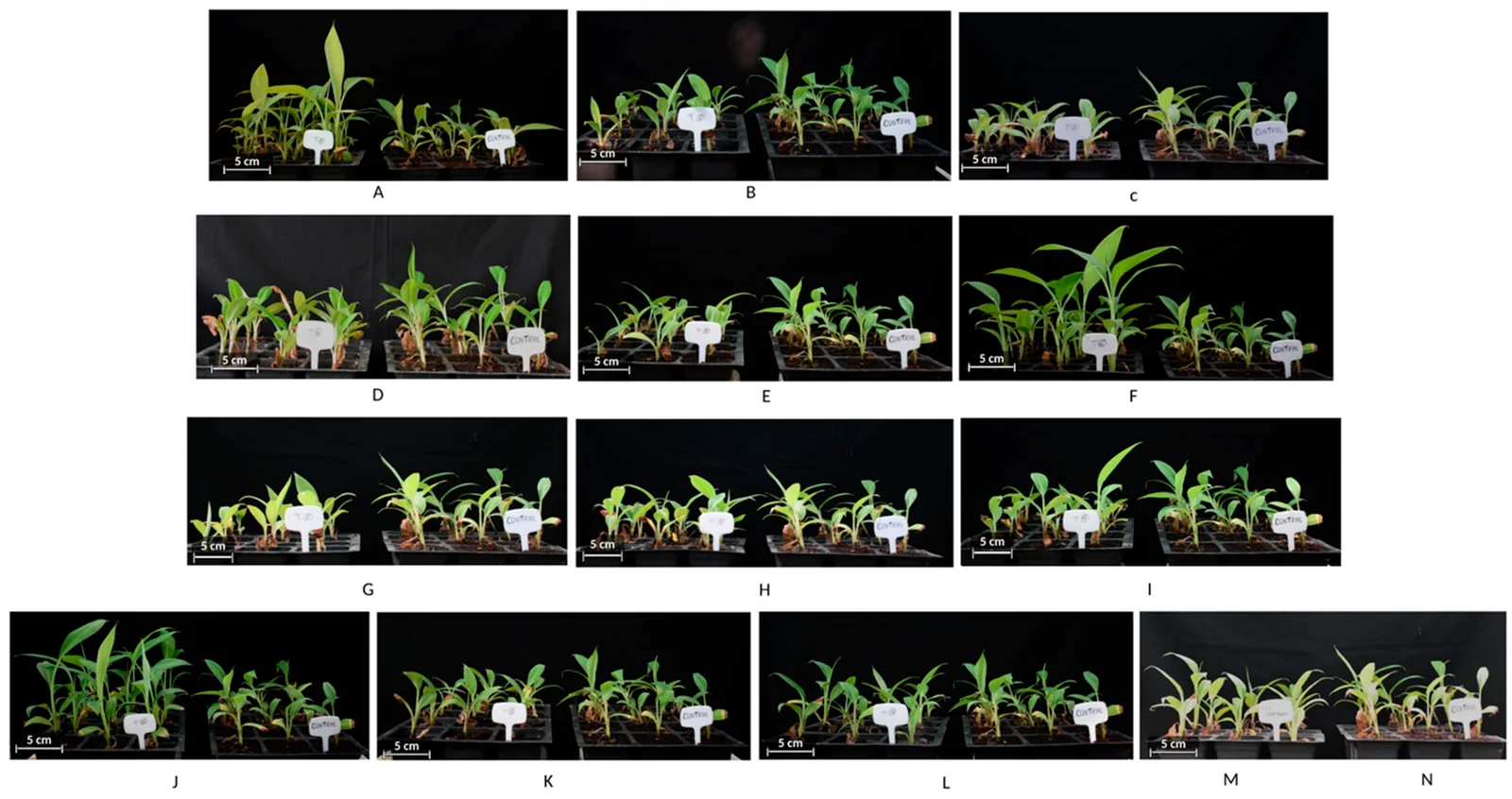
Shoot phenology of plants treated with endophytic bacteria isolated from resistant cultivar compared with uninoculated control; (**A**) *Bacillus subtilis* strain RoC1; (**B**) *Bacillus subtilis* strain RoCo2; (**C**) *Bacillus inaquosorum* strain RoCo3; (**D**) *Bacillus velezensis* strain RoCo4; (**E**) *Stenotrophomonas maltophilia* strain RoCo6; (**F**) *Halomonas* sp. strain RoRo2; (**G**) *Bacillus velezensis* strain RoRo2; (**H**) *Bacillus cereus* strain RoRo15; (**I**) *Stenotrophomonas pavanii* strain RoRo16; (**J**) *Stenotrophomonas maltophilia* strain RoRo17; (**K**) *Bacillus subtilis* strain RoPs8; (**L**) *Pseudomonas putida* strain RoPs13. (**M**) Positive Control; (**N**) Negative Control. The same negative control (**N**) plants were used across all treatments to enable a consistent reference for fair visual comparison.

Significant differences in morphological parameters were observed among the treatments in the pot study. The plants treated with *B. subtilis* strain RoC1 recorded the highest root length (1013.37 cm), root volume (16.47 cm^3^), root weight (8.36 g), and root diameter (2.76 mm) (Figure 7), while also promoting pseudostem height (21.48 cm) and number of leaves (5.62). It was followed by *Halomonas* sp. strain RoRo2, which recorded the highest projected root area (74.97 cm^2^), root surface area (237.51 cm^2^), pseudostem height (23.61 cm), number of leaves (5.78), and leaf area (97.87 cm^2^) (Figure 8). *Stenotrophomonas pavanii* strain RoRo16 recorded the shortest interval between successive leaf appearances (5.25 days), indicating the fastest rate of leaf initiation among all treatments, followed by *Stenotrophomonas maltophilia* strain RoCo6 (5.49 days) and *B. subtilis* strain RoC1 (5.84 days). The positive control recorded the significantly lowest growth promotion, with the lowest root length (154.72 cm), root volume (2.64 cm^3^), root weight (3.22 g), and root diameter (1.00 mm) (Table 4a,b).

**Figure 9:**
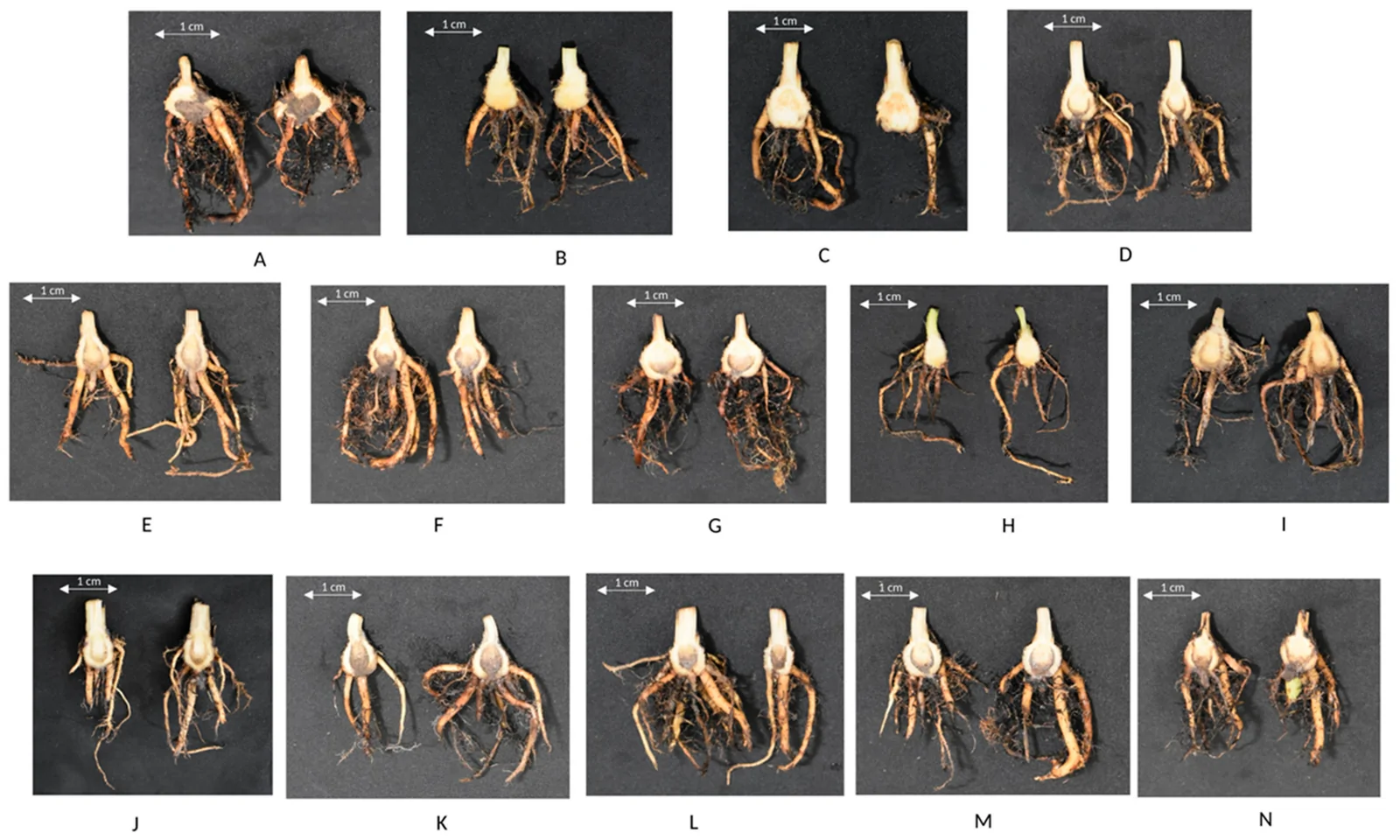
Plants treated with endophytic bacteria isolated from resistant cultivar showing internal *Foc* symptoms, (**A**) Positive Control; (**B**) Negative Control; (**C**) *Bacillus subtilis* strain RoC1; (**D**) *Bacillus subtilis* strain RoCo2; (**E**) *Bacillus inaquosorum* strain RoCo3; (**F**) *Bacillus velezensis* strain RoCo4; (**G**) *Stenotrophomonas maltophilia* strain RoCo6; (**H**) *Halomonas* sp. strain RoRo2; (**I**) *Bacillus velezensis* strain RoRo2; (**J**) *Bacillus cereus* strain RoRo15; (**K**) *Stenotrophomonas pavanii* strain RoRo16; (**L**) *Stenotrophomonas maltophilia* strain RoRo17; (**M**) *Bacillus subtilis* strain RoPs8; (**N**) *Pseudomonas putida* strain RoPs13.

Among all the treatments, *B. subtilis* strain RoC1 and *Halomonas* sp. strain RoRo2 recorded the lowest DI (20%), on par with the negative control, indicating significantly higher disease suppression (Figure 9). *Bacillus cereus* strain RoRo15 exhibited a DI of 40%. *Bacillus subtilis* strain RoCo2, *B. subtilis* subsp. *inaquosorum* strain RoCo3, and *B. subtilis* strain RoPs8 exhibited a DI of 46.67%.

### 3.4. GC-MS Analysis

**Figure 10:**
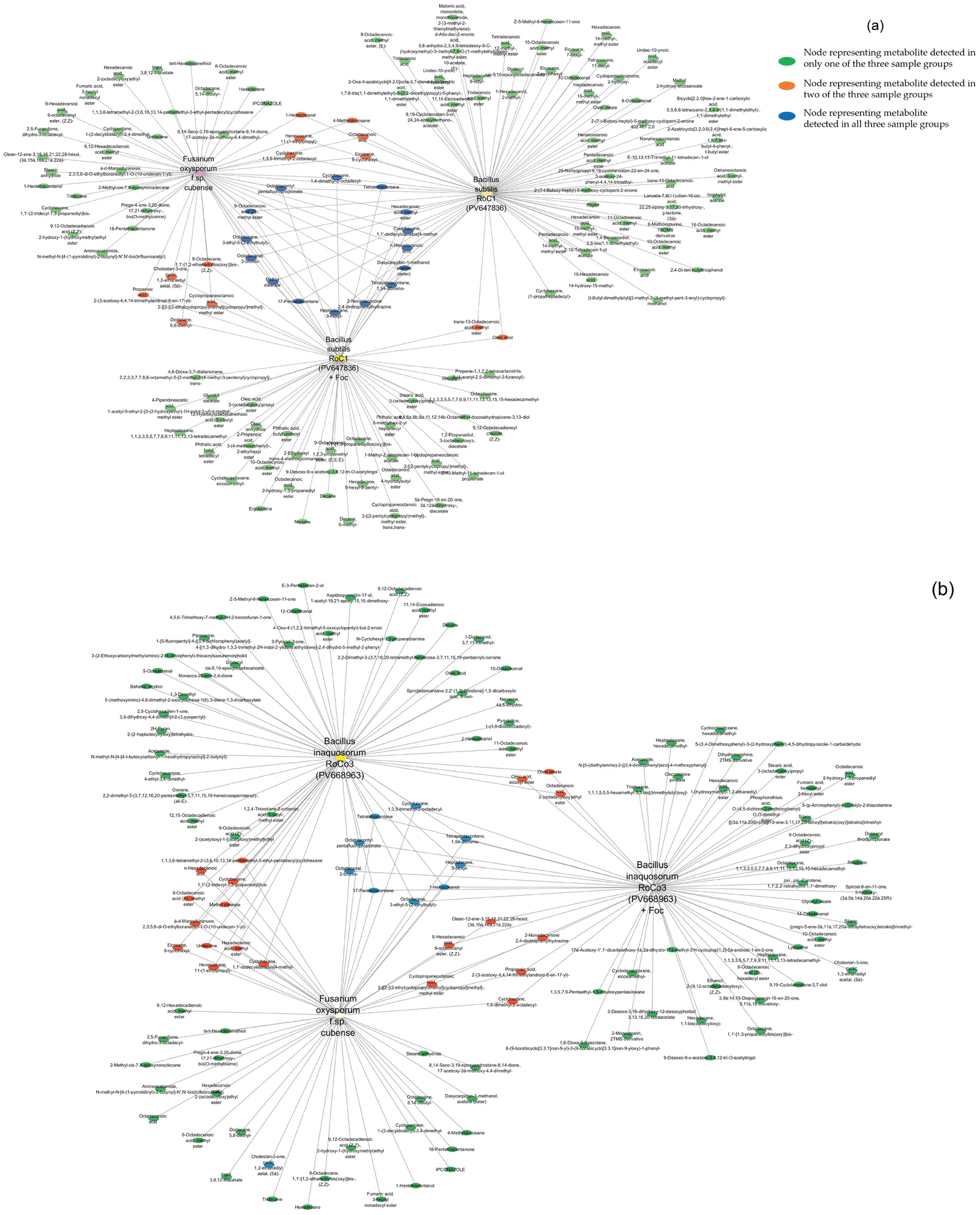

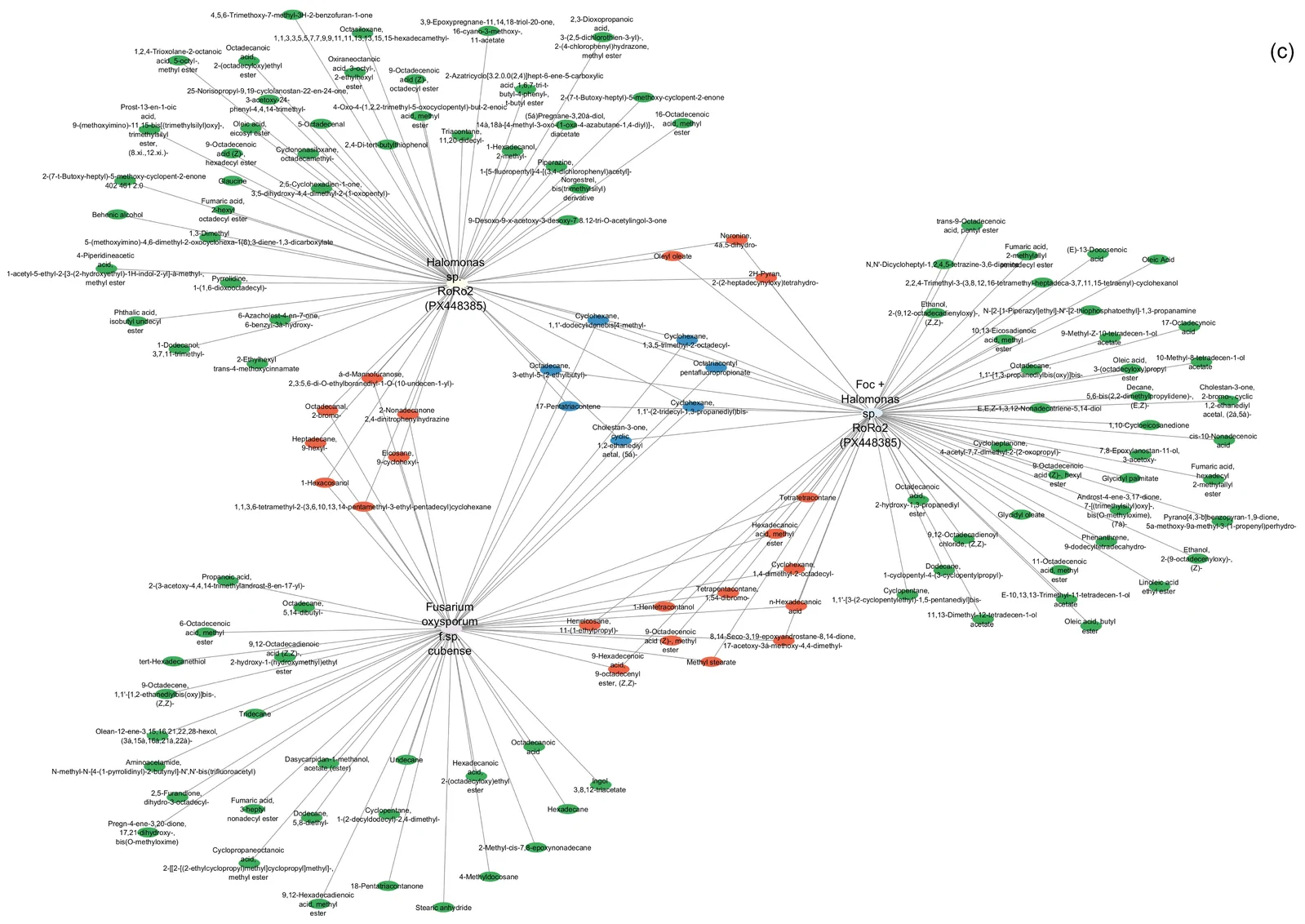
Bipartite network showing metabolites identified in (**a**) *Fusarium oxysporum* f. sp. *cubense* (*Foc*), Bacillus subtilis RoC1 alone and B. subtilis RoC1 + Foc dual-culture, (**b**) *Foc, B. inaquosorum* RoCo3 alone, and *B. inaquosorum* RoCo3 + *Foc*; (**c**) *Foc, Halomonas* sp. RoRo2 alone, and *Halomonas* sp. RoRo2 + *Foc*. Each node represents an individual compound, and edges connect a treatment to every compound detected in it. Node colour indicates the degree of sharing among treatments: green nodes represent metabolites unique to one of the three sample groups, orange nodes represent metabolite shared by two of the three sample groups, blue nodes represent metabolite shared by all three sample groups. Node position reflects connectivity, with shared metabolites drawn centrally between the treatments in which they occur.

GC-MS profiling of secondary metabolites from three endophytic bacterial strains was carried out under three treatment conditions: (i) the endophytic bacteria grown alone (axenic culture), (ii) the endophytic bacteria grown in dual culture (in vitro confrontation) with *Foc*, and (iii) *Foc* grown alone (axenic culture), with the axenic cultures serving as controls to enable comparative identification of metabolites induced during the pathogen-antagonist interaction. This revealed a diverse repertoire of bioactive metabolites, encompassing fatty acid esters, steroids, terpenoids, siloxanes, and nitrogenous compounds, many of which are implicated in antifungal and biotic stress resilience mechanisms (Figure 10). The metabolic profile of *B. subtilis* RoC1, when confronted with *Foc*, exhibited predominant compounds such as 4-piperidineacetic acid, 1-acetyl-5-ethyl-2-[3-(2-hydroxyethyl)-1H-indol-2-yl]-α-methyl-, methyl ester; 12-hydroxyoctadecanethioic acid, S-t-butyl ester; 5α-pregn-16-en-20-one, 3α,12α-dihydroxy-, diacetate; 4,4,6a,6b,8a,11,12,14b-octamethyl-docosahydropicene-3,13-diol; octadecanoic acid, 4-hydroxybutyl ester; glycidol stearate; oleic anhydride; and ergotamine. The presence of these metabolites suggests a complex antimicrobial arsenal involving membrane disruptive and cytotoxic mechanisms.

The metabolic fingerprint of *B. inaquosorum* RoCo3 was characterized by long-chain unsaturated fatty acid methyl esters such as 3-allyloxy-1,2-propanediol, methyl cis-10-pentadecenoate, methyl cis-13,16-docosadienate, methyl cis-11,14-icosadienoate, methyl cis-4,7,10,13,16,19-docosahexaenoate, and methyl oleate. Additional compounds including arachidonic acid-TMS, linoleic acid-TMS, palmitoleic acid-TMS, elaidic acid-TMS, and biotin-3TMS were also detected. Amino acid derivatives such as proline-2TMS, niacinamide-TMS and spermine-6TMS indicate the involvement of nitrogen rich metabolites contributing to antifungal defense and stress regulation.

The GC-MS spectrum of *Halomonas* sp. RoRo2 revealed major metabolites including oleyl oleate, ψ,ψ-carotene 1,1′,2,2′-tetrahydro-1,1′-dimethoxy-, 4-piperidineacetic acid, 5-ethylidene-2-[3-(2-hydroxyethyl)-1H-indol-2-yl]-α-methylene-, methyl ester, 2-eicosanol, clocortolone pivalate, and 1,2-propanediol, 3-(octadecyloxy)-, diacetate. The strain also produced methyl glycocholate (3TMS derivative), chromone (5-hydroxy-6,7,8-trimethoxy-2,3-dimethyl-), glycyl-L-histidyl-L-lysine acetate, fumaric acid, cycloheptyl hexadecyl ester, decyl oleate, pseudosarsasapogenin-5,20-dien methyl ether, and 9-octadecenoic acid (Z)-, 2,3-dihydroxypropyl ester.

## 4. Discussion

*Fusarium* wilt of banana (*Musa* spp.) is one of the most destructive soil-borne diseases worldwide. Despite long-standing efforts to control it through chemical and breeding-based approaches, these attempts have remained largely unsuccessful. But one promising avenue is the exploitation of endophytic bacteria found in resistant wild cultivars, which are known to possess antifungal properties and can help induce host defense responses [21].

Our research revealed that *Bacillus* isolates consistently exhibited strong antifungal activity. Several studies have similarly reported *Bacillus* strains as promising biocontrol agents against a wide range of plant pathogens [22,23,24]. One of the most compelling attributes of *Bacillus* spp. contributing to their biocontrol potential is their remarkable capacity to synthesize a diverse array of antibiotic compounds. These metabolites not only exhibit broad spectrum antimicrobial activity but also influence microbial adhesion, colonization dynamics, and the persistence of *Bacillus* cells within their ecological niche [25]. The mechanisms underlying the observed antifungal activity are complex and involve direct antagonism. *B. licheniformis* is well known to secrete a diverse array of extracellular proteases capable of degrading key proteins and cell wall components of fungal pathogens, thereby directly impairing their pathogenic activity [26,27]. Overall, the results indicate that endophytes isolated from different plant parts possess different antagonistic potential. Corm-associated endophytes produced antifungal metabolites with membrane-disruptive and cytotoxic activities, whereas root-associated endophytes contributed to a modulatory and protective mode of action, suppressing fungal growth and enhancing rhizosphere defense. Differences in endophytic bacterial taxa have been reported by Ajesh et al. [28] and Liu et al. [29], who found that *Acinetobacter*, *Bacillus*, and *Klebsiella* were dominant in the shoot tips, whereas *Caulobacter*, *Staphylococcus*, and *Comamonas* were dominant in the roots of banana. This analysis of the endophytic bacterial communities across various banana cultivars strongly supports the functional role of specific taxa in determining host health. Across different geographical regions, researchers consistently identified Bacillus, Pseudomonas, and Enterobacter as abundant genera within the healthy banana phytobiome [30,31,32]. Hardoim et al. [33] posited that the alpha and beta diversity of the endophytic community can significantly influence the host plant’s genetic makeup, physiology, and morphology. Therefore, variations in endophytic diversity are likely key contributors to differences in disease resistance and phenotypic traits observed among banana cultivars.

The functional profiling sharply delineated the biocontrol potential between the resistant cv. Rose (AB) and susceptible cv. Karpooravalli (ABB). The strong antifungal activity demonstrated by endophytes from ‘Rose (AB)’ suggests this cultivar maintains a richer, more active reservoir of antagonistic bacteria, which contributes to its enhanced resistance. Conversely, the absence of comparable antifungal activity among cultivable endophytes from “Karpooravalli (ABB)” points to a weaker native endophytic defense system in the susceptible cultivars. This pattern mirrors observations in other agricultural crops as well, such as bean cultivars, where susceptible varieties typically harbor less complex bacterial communities and resistant varieties have a highly diverse and rich microbiome [34]. While antagonistic endophytes are indeed effective, studies isolating only a few such organisms from numerous total isolates in *Foc*-infested fields highlight that their natural prevalence is often low [35,36]. Therefore, extensive exploration, targeted bioprospecting, and high throughput screening approaches are required to uncover a wider diversity of beneficial strains. Such efforts are pivotal for developing multi-strain microbial consortia which will deliver a comprehensive and long-term protection against diverse pathogens.

A major outcome of this investigation is the pivotal role of the genus *Bacillus* and *Halomonas* in the biological control of *Foc* in banana. The growth responses we observed are consistent with the plant growth-promoting (PGP) mechanisms widely documented in endophytic *Bacillus* sp., notably biological nitrogen fixation, phosphate solubilization, and indole-3-acetic acid (IAA) biosynthesis. *Halomonas* spp. has also been reported to promote plant growth even under saline stress through the production of exopolysaccharides, which sequester arsenic and thereby restore essential phytohormone (IAA, JA, SA) [37]. Auxin-mediated stimulation of lateral root and root-hair proliferation increased root length, surface area, and volume, thereby enlarging the absorptive interface for water and mineral uptake. Simultaneously, the increase in pseudostem height and leaf area expanded the photosynthetic source, improving assimilate supply to developing tissues. The significant increase in leaf number and leaf area further points to cytokinin-mediated effects on shoot development and overall plant vigor, complementing the auxin-driven gains in root architecture [38,39]. Such vigor is likely to improve the host’s capacity to tolerate and mitigate for *Foc*-induced vascular damage. The markedly inferior performance of the inoculated (positive) control across these parameters reinforces the protective contribution of the introduced endophytes. *B. velezensis*, for instance, demonstrated potent antagonism against *Foc*, inflicting pronounced morphological damage and achieving a notable inhibition rate of 75.43% [40]. SEM analysis confirmed the organism’s ability to colonize host tissues, which, following biopriming, successfully protected tissue culture plantlets from *Foc* infection. Endophytic colonization has been directly demonstrated by Sun et al. [41] who reported that *B. subtilis* strain TR21 increased root length, formed biofilms on root surfaces, and triggered induced systemic resistance (ISR) in the host, in addition to directly inhibiting fungal pathogens. *Bacillus amyloliquefaciens*, isolated from banana, was shown to promote plant growth and inhibit *Foc* through both antimicrobial peptide and VOC production. *Bacillus halotolerans* isolates also displayed key plant growth promoting traits, including nitrogen fixation, phosphate solubilization, and auxin production, alongside enhanced tolerance to heavy metals and xenobiotics.

Comparative genomics successfully characterized the open pangenome of *B. halotolerans*, pinpointing functional genes responsible for these beneficial traits. The identification of the cheA gene in *Halomonas* species is notable, as CheA serves as the central kinase of the chemotaxis signaling pathway, enabling the bacterium to detect and respond to root-derived chemo effectors [42]. Its interaction with chemoreceptor proteins points to an enhanced capacity to sense the diverse chemical cues exuded by host roots, particularly those of resistant cultivars, thereby promoting efficient root colonization and niche establishment by antagonistic bacteria [43]. This explains the observed differences in microbial composition between resistant and susceptible plants; resistant cultivars appear to preferentially recruit chemotaxis-competent bacteria through metabolite signaling. In this way, chemotaxis-mediated host–microbe crosstalk acts as a key determinant in shaping and enriching the beneficial bacterial community.

Recent studies have highlighted that fatty acids and their derivatives, which constitute a major component of the identified bioactive molecules, such as nonanol [44] and octadecadienoic acid, are recognized for their strong antimicrobial activity, acting primarily through membrane disruption that weakens cell integrity and causes leakage of cellular contents [45]. Consistent with this, volatile organic compounds (VOCs) secreted by *Stenotrophomonas geniculata* achieved 100% inhibition of *Fusarium* mycelial growth in Lanzhou lily bulbs, with SEM analysis revealing severe morphological distortions of hyphae indicative of structural degradation [46]. Similarly, the marine bacterium *Stenotrophomonas rhizophila* has shown promising biocontrol efficacy against *Fusarium proliferatum* in muskmelon, further supporting the role of VOC-mediated antagonism [47].

Disease suppression by these endophytes involves complex mechanisms, encompassing direct antagonism through antimicrobial metabolites, competition for iron, biofilm formation on root surfaces that creates a protective barrier, and activation of host defense responses [48]. The presence of antibiotic biosynthesis genes in several *Bacillus* strains contributes to the biocontrol of plant pathogens [49,50], such as upregulation of the bamB gene, which drives the biosynthetic pathway of bacillomycin, a key member of the iturin family. Lipopeptides such as iturin A and surfactin, secreted by *B. subtilis*, act largely through pore formation in fungal membranes [51,52,53], while fengycin disrupts fungal cell wall and membrane integrity, often working synergistically with iturin to intensify antifungal activity. Glycerol- and glycidol-derived esters contribute by modifying membrane fluidity and inhibiting spore germination and help in synthesis of fengycin [54]. The application of these compounds has proved to induce severe morphological distortions in fungal hyphae, such as swelling, vacuolization, and lysis, leading to profound growth inhibition of the pathogen [55]. Fengycin primarily targets and disrupts the plasma membranes of fungal cells, and disruption leads to morphological changes in fungal hyphae, causing them to bulge and curl [22]. These findings are consistent with our study where bulging and curling of fungal mycelium was observed in agar well assay of *B. subtilis* RoC1, *Halomonas* sp. RoRo2, *B. inaquosorum* RoCo3 isolates. Fengycin like hydroxy fatty acid derivatives were also detected among the metabolites produced by the antagonistic *Bacillus* strains, including 12-hydroxyoctadecanethioic acid, S-t-butyl ester; octadecanoic acid, 4-hydroxybutyl ester; and octadecanoic acid, 2-hydroxy-1,3-propanediyl ester. These hydroxylated fatty acids are known to enhance membrane permeability and contribute to biosurfactant activity, thereby disrupting fungal cell integrity and inhibiting hyphal growth. This metabolite diversity also extends to cyclopropane containing fatty acids, which interfere with essential fungal lipid metabolism, while alkaloids target fungal signaling and cell wall processes. This repertoire further includes polyketides such as difficidin and macrolactin, produced by *B. velezensis*, which exert broad antimicrobial effects by inhibiting key enzymatic processes [56].

Oleic acid, 3-(octadecyloxy) propyl ester was reported to exhibit strong antifungal activity against major plant pathogens such as *Colletotrichum fulcatum*, *Fusarium oxysporum*, and *Rhizoctonia solani* [57]. The presence of this compound in the extract suggests that it may play a key role in suppressing fungal growth by interfering with the integrity of the fungal cell membrane. Similarly, stearic acid, 3-(octadecyloxy) propyl ester, has also been reported to possess potent fungicidal properties against both plant and human fungal pathogens [57]. The octadecyloxy side chain of stearic acid is thought to disrupt the lipid bilayer of fungal membranes, leading to leakage of intracellular contents and eventual cell lysis, thereby contributing to its strong antifungal efficacy. These findings are consistent with those of Mousa et al. [58], who reported that *Halomonas* produced hexadecanoic acid, methyl ester (33.22%) and 9-octadecenoic acid, both of which are recognized for their biosurfactant and antimicrobial properties and thus contribute to suppression of the *Fusarium* pathogen. Other related long-chain fatty acid esters such as octadecanoic acid methyl ester and 2-hydroxy-1,3-propanediyl ester have been documented for their antioxidant and antimicrobial activities [59], supporting the multifunctional bioactivity of these lipid derivatives. Monoacylglycerols and their analogues further enhance this effect, as they can interact directly with fungal membranes through hydrogen bonding and hydrophobic interactions, resulting in cell wall destabilization and membrane rupture [60]. These mechanisms collectively indicate that fatty acid derivatives, especially those with long alkyl or acyloxy substituents, can serve as potent membrane-active antifungal agents.

In addition to these, Piperidine acetic acid, 1-acetyl-5-ethyl-2-[3-(2-hydroxyethyl)-1H-indol-2-yl]-methyl, methyl ester has been previously reported to display antifungal activity [61,62]. Its presence in the extract highlights the diverse chemical nature of antifungal constituents, extending beyond lipid derivatives to include nitrogen containing heterocycles with potential bioactivity. Morphological studies on eicosane-treated fungal cells revealed pronounced structural damage, including distorted hyphae, flaccid mycelia, and collapsed spores, in contrast to the turgid and intact structures observed in untreated control [63,64]. Such observations suggest that eicosane disrupts fungal cellular integrity, possibly through hydrophobic interactions affecting membrane fluidity and permeability.

Compounds belonging to the acetylenic fatty acid class, such as 6-heptadecynoic acid and 17-octadecynoic acid, are also known for their significant antifungal potency [65]. Acetylenic acids are characterized by triple bonds within their carbon chain, which confer unique reactivity and facilitate interactions with cellular enzymes and membrane lipids, resulting in strong inhibitory effects against fungal pathogens. Additionally, fumaric acid and its derivatives have been recognized for their antifungal potential, along with oleic acid, which has demonstrated inhibitory activity against *Fusarium oxysporum* [66]. The combined occurrence of these compounds in the extract indicates a synergistic antifungal profile, where both unsaturated fatty acids and organic acid derivatives contribute to overall activity through multiple biochemical pathways.

Taken together, these findings strongly suggest that the identified metabolites, particularly the long-chain fatty acid esters, acetylenic acids, and nitrogenous compounds, endow the extract with potent antifungal properties. Their mechanisms involve disruption of membrane integrity, interference with essential enzymatic functions, and induction of oxidative stress within fungal cells. The diverse chemical nature and complementary modes of action of these metabolites highlight their potential as natural antifungal agents. The synergistic action of direct antagonism, metabolite-mediated inhibition, growth promotion, and putative host-defense induction by *Bacillus* sp. and *Halomonas* sp. establishes these two strains as the most promising candidates for integrated management of *Fusarium* wilt in banana cultivation.

## 5. Conclusions

The present study elucidates the biocontrol potential of endophytic bacterial species against *Foc*, emphasizing the influence of host cultivar on endophyte efficacy. The antagonistic endophytes isolated from the resistant wild cultivar were consistently the most effective across the in vitro and in planta assays, and their activity was multifactorial, combining direct antagonism, the production of membrane-disruptive secondary metabolites, and the promotion of plant growth. Because suppression of *Foc* rests on several independent mechanisms rather than a single mode of action, the protection conferred is likely to be more durable and less prone to pathogen adaptation than conventional single-target chemical control. The close concordance between the metabolite profiles characterized in vitro and the disease suppression observed in the pot experiment further indicates that metabolite profiling provides supporting evidence for the disease-suppressive activity of effective biocontrol strains against *Foc*.

The identification and characterization of these metabolically versatile endophytes represent a pivotal advancement toward developing efficient and environmentally compatible bioinoculants. Moreover, the strategic formulation of microbial consortia with complementary biochemical and functional traits holds promise for improving product stability, enhancing field performance, and ensuring consistent efficacy across variable agroecological environments. Such integrative approaches will provide a sustainable and reliable alternative to conventional chemical formulations.

## Figures and Tables

**Table 1:** Composition of the PCR reaction mixture used for 16S rRNA amplification.

PCR Component	Amount (per 25 µL Reaction)
PCR buffer	1×
dNTPs	200 µM
Primers 27F and 1492R (each)	0.5 µM
MgCl_2_	1.5 mM
Taq DNA polymerase	1 U
Genomic DNA (template)	50 ng

**Table 2:** Disease severity scale for *Fusarium* wilt of banana.

Grade 1	No symptoms
Grade 2	10–20% initial corm discoloration
Grade 3	20–40% slight discoloration of the corm
Grade 4	40–60% discoloration of the corm
Grade 5	60–80% complete discoloration of the corm

**Table 3:** Endophytic bacterial isolates from the resistant (Rose, AB) and susceptible (Karpooravalli, ABB) banana cultivars.

Cultivar	Source Tissue	Isolate (Closest Match, 16S rRNA)	GenBank Accession
Resistant (Rose, AB)	Corm	*Bacillus subtilis* strain RoC1	PV647836
	Corm	*Bacillus subtilis* strain RoCo2	PV668949
	Corm	*Bacillus inaquosorum* strain RoCo3	PV668963
	Corm	*Bacillus velezensis* strain RoCo4	PV668996
	Corm	*Stenotrophomonas maltophilia* strain RoCo6	PV669005
	Root	*Halomonas* sp. strain RoRo2	PX448385
	Root	*Bacillus velezensis* strain RoRo2	PV670015
	Root	*Bacillus cereus* strain RoRo15	PV670002
	Root	*Stenotrophomonas pavanii* strain RoRo16	PV670050
	Root	*Stenotrophomonas maltophilia* strain RoRo17	PV670070
	Pseudostem	*Bacillus subtilis* strain RoPs8	PV670073
	Pseudostem	*Pseudomonas putida* strain RoPs13	PV678980
Susceptible (Karpooravalli, ABB)	Root	*Brevibacterium casei* strain KaRo1	PV668918
	Root	*Rhodocyclaceae bacterium* strain KaRo2	PV682272
	Root	*Rhizobium pusense* strain KaRo3	PV682315
	Corm	*Bacillus subtilis* subsp. *stercoris* strain KaCo4	PV687459
	Corm	*Bacillus subtilis* strain KaCo6	PV687502
	Corm	*Bacillus zhangzhouensis* strain KaCo7	PV687508
	Corm	*Brevibacterium iodinum* strain KaCo8	PV687509
	Corm	*Bacillus licheniformis* strain KaCo9	PV687510
	Pseudostem	*Bacillus cereus* strain KaPs1	PV687495
	Pseudostem	*Pseudomonas parafulva* strain KaPs2	PV687500

**Table 4:** (**a**) Root Morphology evaluation of banana plants treated with antagonist bacterial endophytes and infected with *Foc.* (**b**) Shoot morphology evaluation and disease index of banana plants treated with antagonist bacterial endophytes and infected with *Foc*.

**(a)**						
**Treatment**	**Root Length (cm)**	**Projected Root Area (cm^2^)**	**Root Surface Area (cm^2^)**	**Root Diameter (mm)**	**Root Volume (cm^3^)**	**Root Weight (g)**
Positive Control	154.72 ^m^	52.38 ^f^	151.06 ^g^	1.00 ^j^	2.64 ^k^	3.22 ^k^
Negative Control	275.06 ^l^	28.80 ^i^	108.63 ^i^	0.94 ^j^	12.50 ^e^	4.14 ^h^
*Bacillus subtilis* strain RoC1 (PV647836)	1013.37 ^a^	74.86 ^a^	209.24 ^b^	2.76 ^a^	16.47 ^a^	8.36 ^a^
*Bacillus subtilis* strain RoCo2 (PV668949)	374.79 ^h^	52.04 ^f^	155.27 ^fg^	1.16 ^h^	12.22 ^e^	4.26 ^gh^
*Bacillus inaquosorum* strain RoCo3 (PV668963)	786.20 ^b^	67.05 ^c^	194.33 ^c^	2.45 ^c^	14.63 ^c^	7.55 ^c^
*Bacillus velezensis* strain RoCo4 (PV668996)	592.07 ^d^	44.80 ^g^	165.53 ^d^	1.33 ^g^	13.53 ^d^	4.79 ^e^
*Stenotrophomonas maltophilia* strain RoCo6 (PV669005)	294.16 ^k^	29.56 ^i^	110.38 ^i^	1.07 ^i^	12.39 ^e^	4.30 ^g^
*Halomonas* sp. strain RoRo2 (PX448385)	743.61 ^c^	74.97 ^a^	237.51 ^a^	2.24 ^d^	15.23 ^b^	7.98 ^b^
*Bacillus velezensis* strain RoRo2 (PV670015)	547.72 ^e^	53.23 ^f^	137.06 ^h^	1.50 ^f^	11.53 ^f^	3.41 ^j^
*Bacillus cereus* strain RoRo15 (PV670002)	521.19 ^f^	55.03 ^e^	111.65 ^i^	1.12 ^hi^	10.46 ^h^	3.75 ^i^
*Stenotrophomonas pavanii* strain RoRo16 (PV670050)	381.84 ^g^	58.94 ^d^	154.51 ^g^	2.04 ^e^	10.91 ^g^	3.18 ^k^
*Stenotrophomonas maltophilia* strain RoRo17 (PV670070)	783.22 ^b^	69.99 ^b^	190.69 ^c^	2.56 ^b^	14.73 ^c^	5.06 ^d^
*Bacillus subtilis* strain RoPs8 (PV670073)	321.78 ^j^	37.25 ^h^	159.82 ^ef^	2.39 ^c^	9.06 ^i^	3.86 ^i^
*Pseudomonas putida* strain RoPs13 (PV678980)	327.33 ^i^	38.32 ^h^	160.94 ^de^	2.04 ^e^	8.31 ^j^	4.61 ^f^
SE ±	1.71	0.63	1.78	0.02	0.12	0.05
CV%	1.01	3.58	3.33	3.95	3.12	2.83
**(b)**						
**Treatment**	**Pseudostem Height (cm)**	**No. of Leaves**	**Leaf Area (cm^2^)**	**Pseudostem Girth (cm)**	**Phyllochron (days)**	**Disease Index (%)**
Positive Control	9.29 ^d^	3.61 ^f^	19.68 ^e^	1.74 ^a^	8.05 ^b^	93.33 ^a^
Negative Control	6.89 ^hi^	3.71 ^e^	16.18 ^f^	1.19 ^ef^	5.32 ^g^	20.00 ^g^
*Bacillus subtilis* strain RoC1 (PV647836)	21.48 ^b^	5.62 ^b^	57.08 ^c^	1.15 ^f^	5.84 ^e^	20.00 ^g^
*Bacillus subtilis* strain RoCo2 (PV668949)	8.93 ^e^	3.31 ^j^	19.89 ^e^	1.28 ^d^	8.37 ^a^	46.67 ^e^
*Bacillus inaquosorum* strain RoCo3 (PV668963)	7.40 ^g^	3.62 ^f^	12.94 ^h^	1.42 ^c^	7.97 ^b^	46.67 ^e^
*Bacillus velezensis* strain RoCo4 (PV668996)	6.30 ^j^	3.55 ^g^	14.58 ^g^	0.96 ^h^	8.05 ^b^	60.00 ^c^
*Stenotrophomonas maltophilia* strain RoCo6 (PV669005)	6.85 ^i^	3.81 ^d^	16.43 ^f^	1.29 ^d^	5.49 ^f^	53.33 ^d^
*Halomonas* sp. strain RoRo2 (PX448385)	23.61 ^a^	5.78 ^a^	97.87 ^a^	1.03 ^g^	6.27 ^d^	20.00 ^g^
*Bacillus velezensis* strain RoRo2 (PV670015)	6.04 ^k^	3.46 ^h^	15.47 ^fg^	1.00 ^gh^	7.93 ^bc^	53.33 ^d^
*Bacillus cereus* strain RoRo15 (PV670002)	8.42 ^f^	3.61 ^f^	15.56 ^fg^	1.32 ^d^	8.28 ^a^	40.00 ^f^
*Stenotrophomonas pavanii* strain RoRo16 (PV670050)	7.10 ^h^	3.40 ^i^	16.07 ^f^	1.60 ^b^	5.25 ^g^	73.33 ^b^
*Stenotrophomonas maltophilia* strain RoRo17 (PV670070)	21.46 ^b^	5.57 ^c^	78.98 ^b^	1.22 ^e^	7.85 ^c^	60.00 ^c^
*Bacillus subtilis* strain RoPs8 (PV670073)	9.43 ^d^	3.62 ^f^	15.7 ^f^	1.18 ^ef^	7.83 ^c^	46.67 ^e^
*Pseudomonas putida* strain RoPs13 (PV678980)	10.04 ^c^	3.35 ^j^	23.77 ^d^	1.00 ^gh^	8.28 ^a^	53.33 ^d^
SE ±	0.08	0.01	0.33	0.02	0.04	0.80
CV%	2.29	1.21	3.33	3.64	1.83	2.81

The lowercase superscript letters in the table denote statistically significant differences (Duncan’s Multiple Range Test (α = 0.05)).

## Data Availability

The original contributions presented in this study are included in the article/Supplementary Materials. Further inquiries can be directed to the corresponding author.

## Supplementary Materials

The following supporting information can be downloaded at: https://www.mdpi.com/article/10.3390/pathogens15080864/s1, Supplementary Figure S1. Pseudostem, corm, and root of banana (*Musa* spp.) used as sources for endophytic bacterial isolation.

## References

[1] Bebber D.P. (2023). The long road to a sustainable banana trade. Plants People Planet.

[2] APEDA (2025). Monthly Dashboard Banana. https://apeda.gov.in/sites/default/files/2025-10/MIC_Monthly_dashboard_Banana_22092025.pdf.

[3] Department of Agriculture & Farmers Welfare (MoA & FW) (2025). Horticulture Statistics. https://agriwelfare.gov.in/en/StatHortEst.

[4] Pegg K.G., Coates L.M., O’Neill W.T., Turner D.W. (2019). The epidemiology of *Fusarium* wilt of banana. Front. Plant Sci..

[5] Ding Z., Yang L., Wang G., Guo L., Liu L., Wang J., Huang J. (2018). Fusaric acid is a virulence factor of *Fusarium oxysporum* f. sp. *cubense* on banana plantlets. Trop. Plant Pathol..

[6] Bermúdez-Caraballoso I., Cruz-Martín M., Concepción-Hernández M. (2020). Biotechnological tools for the development of *Foc* TR4-resistant or -tolerant *Musa* spp. cultivars. Agricultural, Forestry and Bioindustry Biotechnology and Biodiscovery.

[7] Vimal S.R., Singh J.S., Kumar A., Prasad S.M. (2024). The plant endomicrobiome: Structure and strategies to produce stress resilient future crop. Curr. Res. Microb. Sci..

[8] Kumar V., Nautiyal C.S. (2023). Endophytes modulate plant genes: Present status and future perspectives. Curr. Microbiol..

[9] Kavino M., Jegan P., Nakkeeran S., Raveendran M., Chetry S. (2025). Bio hardening of banana (*Musa* spp.) with bacterial endophytes to enhance resistance against *Fusarium* wilt. Rhizosphere.

[10] Kavino M., Manoranjitham S.K., Kumar N., Vijayakumar R.M. (2016). Plant growth stimulation and bio control of *Fusarium* wilt (*Fusarium oxysporum* f. sp. *cubense*) by co inoculation of banana (*Musa* spp.) plantlets with PGPR and endophytes. Recent Trends in PGPR Research for Sustainable Crop Productivity.

[11] Kavino M., Manoranjitham S.K. (2018). In vitro bacterization of banana (*Musa* spp.) with native endophytic and rhizospheric bacterial isolates: Novel ways to combat *Fusarium* wilt. Eur. J. Plant Pathol..

[12] Singh S., Aghdam S.A., Lahowetz R.M., Brown A.M. (2023). Metapangenomics of wild and cultivated banana microbiome reveals a plethora of host-associated protective functions. Environ. Microbiome.

[13] De Lamo F.J., Takken F.L. (2020). Biocontrol by *Fusarium oxysporum* using endophyte-mediated resistance. Front. Plant Sci..

[14] Bacon C.W., Hinton D.M. (2002). Endophytic and biological control potential of *Bacillus mojavensis* and related species. Biol. Control.

[15] Khamna S., Yokota A., Lumyong S. (2009). Actinomycetes isolated from medicinal plant rhizosphere soils: Diversity and screening of antifungal compounds, indole-3-acetic acid and siderophore production. World J. Microbiol. Biotechnol..

[16] Dennis C., Webster J. (1971). Antagonistic properties of species-groups of *Trichoderma*: I. Production of non-volatile antibiotics. Trans. Br. Mycol. Soc..

[17] Green M.R., Sambrook J. (2018). Isolation and quantification of DNA. Cold Spring Harb. Protoc..

[18] Lane D.J. (1991). 16S/23S rRNA sequencing. Nucleic acid techniques in bacterial systematics. Nucleic Acid Techniques in Bacterial Systematic.

[19] Dita M., Barquero M., Heck D., Mizubuti E.S., Staver C.P. (2018). *Fusarium* wilt of banana: Current knowledge on epidemiology and research needs toward sustainable disease management. Front. Plant Sci..

[20] Shifa H., Gopalakrishnan C., Velazhahan R. (2015). Characterization of antifungal antibiotics produced by *Bacillus subtilis* G-1 antagonistic to *Sclerotium rolfsii*. Biochem. Cell. Arch..

[21] Savani A.K., Bhattacharyya A., Boro R.C., Dinesh K., Swamy Jc N. (2021). Exemplifying endophytes of banana (*Musa paradisiaca*) for their potential role in growth stimulation and management of *Fusarium oxysporum* f. sp. *cubense* causing panama wilt. Folia Microbiol..

[22] Duan Y., Pang Z., Yin S., Xiao W., Hu H., Xie J. (2023). Screening and analysis of antifungal strains *B. subtilis* JF-4 and *B. amylum* JF-5 for the biological control of *Fusarium* wilt of banana. J. Fungi.

[23] Fan H., He P., Xu S., Li S., Wang Y., Zhang W., Li X., Shang H., Zeng L., Zheng S.J. (2023). Banana disease-suppressive soil drives *Bacillus* assembled to defense *Fusarium* wilt of banana. Front. Microbiol..

[24] Win T.T., Bo B., Malec P., Fu P. (2021). The effect of a consortium of *Penicillium* sp. and *Bacillus* spp. in suppressing banana fungal diseases caused by *Fusarium* sp. and *Alternaria* sp. J. Appl. Microbiol..

[25] Stein T. (2005). *Bacillus subtilis* antibiotics: Structures, syntheses and specific functions. Mol. Microbiol..

[26] Liu L., Li X., Li T., Xie Y., Cao Z., Fang P. (2022). Bio-organic fertilizer with *B. subtilis* F2 promotes strawberry plant growth and changes microbial community. J. Soil Sci. Plant Nutr..

[27] Chen J., Deng Y., Yu X., Wu G., Gao Y., Ren A. (2023). *Epichloë* endophyte infection changes the root endosphere microbial community composition of *Leymus chinensis* under both potted and field growth conditions. Microb. Ecol..

[28] Ajesh B.R., Renukadevi P., Saranya N., Vidhyashri N., Varanavasiappan S., Vellaikumar S., Ashraf S., Haripriya S., Raish M., Nakkeeran S. (2025). Genome-wide exploration of beneficial *Bacillus subtilis* isolate from resistant banana cultivar Anaikomban towards the management of *Fusarium* wilt in banana. J. Agric. Food Res..

[29] Liu Y., Zhu A., Tan H., Cao L., Zhang R. (2019). Engineering banana endosphere microbiome to improve *Fusarium* wilt resistance in banana. Microbiome.

[30] Kaushal M., Swennen R., Mahuku G. (2020). Unlocking the microbiome communities of banana (*Musa* spp.) under disease stressed (*Fusarium* wilt) and non-stressed conditions. Microorganisms.

[31] Mohite D.P., Kavino M., Nakkeeran S., Raveendran M., Raghu R., Vethamoni P.I., Saranya N. (2024). Biohardening with endomicrobiome-A novel approach to develop *Fusarium* wilt resistance in banana (*Musa* spp.). Microbe.

[32] Rossmann M., Sarango-Flores S.W., Chiaramonte J.B., Kmit M.C.P., Mendes R. (2017). Plant microbiome: Composition and functions in plant compartments. The Brazilian Microbiome: Current Status and Perspectives.

[33] Hardoim P.R., Andreote F.D., Reinhold-Hurek B., Sessitsch A., van Overbeek L.S., van Elsas J.D. (2011). Rice root-associated bacteria: Insights into community structures across 10 cultivars. FEMS Microbiol. Ecol..

[34] Mendes L.W., Raaijmakers J.M., de Hollander M., Mendes R., Tsai S.M. (2018). Influence of resistance breeding in common bean on rhizosphere microbiome composition and function. ISME J..

[35] Wang B., Yuan J., Zhang J., Shen Z., Zhang M., Li R., Ruan Y., Shen Q. (2013). Effects of novel bioorganic fertilizer produced by *Bacillus amyloliquefaciens* W19 on antagonism of *Fusarium* wilt of banana. Biol. Fertil. Soils.

[36] Souza A., Cruz J.C., Sousa N.R., Procópio A.R.L., Silva G.F. (2014). Endophytic bacteria from banana cultivars and their antifungal activity. Genet. Mol. Res..

[37] Mukherjee P., Mitra A., Roy M. (2019). *Halomonas* rhizobacteria of *Avicennia marina* of Indian Sundarbans promote rice growth under saline and heavy metal stresses through exopolysaccharide production. Front. Microbiol..

[38] Vacheron J., Desbrosses G., Bouffaud M.L., Touraine B., Moënne-Loccoz Y., Muller D., Legendre L., Wisniewski-Dyé F., Prigent-Combaret C. (2013). Plant growth-promoting rhizobacteria and root system functioning. Front. Plant Sci..

[39] Li Y., Shao J., Xie Y., Jia L., Fu Y., Xu Z., Zhang N., Feng H., Xun W., Liu Y. (2021). Volatile compounds from beneficial rhizobacteria *Bacillus* spp. promote periodic lateral root development in *Arabidopsis*. Plant Cell Environ..

[40] Xiang D., Yang X., Liu B., Chu Y., Liu S., Li C. (2023). Bio-priming of banana tissue culture plantlets with endophytic *B. velezensis* EB1 to improve *Fusarium* wilt resistance. Front. Microbiol..

[41] Sun Y., Huang B., Cheng P., Li C., Chen Y., Li Y., Zheng L., Xing J., Dong Z., Yu G. (2022). Endophytic *Bacillus subtilis* TR21 improves banana plant resistance to *Fusarium oxysporum* f. sp. *cubense* and promotes root growth by upregulating the jasmonate and brassinosteroid biosynthesis pathways. Phytopathology.

[42] Balmaceda R.S., Ramos Ricciuti F.E., Redersdorff I.E., Veinticcinque L.M., Studdert C.A., Herrera Seitz M.K. (2022). Chemosensory pathways of *Halomonas titanicae* KHS3 control chemotaxis behavior and biofilm formation. Microbiology.

[43] Sampedro I., Pérez-Mendoza D., Toral L., Palacios E., Arriagada C., Llamas I. (2020). Effects of halophyte root exudates and their components on chemotaxis, biofilm formation and colonization of the halophilic bacterium *Halomonas anticariensis* FP35T. Microorganisms.

[44] Ravi S., Sevugapperumal N., Nallusamy S., Shanmugam H., Mathiyazhagan K., Rangasamy A., Akkanna Subbiah K., Varagur Ganesan M. (2022). Differential bacterial endophytome in Foc-resistant banana cultivar displays enhanced antagonistic activity against *Fusarium oxysporum* f. sp. *cubense* (Foc). Environ. Microbiol..

[45] Slama H.B., Cherif-Silini H., Chenari Bouket A., Qader M., Silini A., Yahiaoui B., Alenezi F.N., Luptakova L., Triki M.A., Vallat A. (2019). Screening for *Fusarium* antagonistic bacteria from contrasting niches designated the endophyte *Bacillus halotolerans* as plant warden against *Fusarium*. Front. Microbiol..

[46] Ling L., Yue R., Wang Y., Feng L., Yang L., Li Y., Mo R., Zhang W., Kong F., Jiang Y. (2025). Volatile organic compounds from *Stenotrophomonas geniculata* J-0 as potential biofumigants manage bulb rot caused by *Fusarium oxysporum* in postharvest Lanzhou lily. World J. Microbiol. Biotechnol..

[47] Rivas-Garcia T., Murillo-Amador B., Nieto-Garibay A., Rincon-Enriquez G., Chiquito-Contreras R.G., Hernandez-Montiel L.G. (2019). Enhanced biocontrol of fruit rot on muskmelon by combination treatment with marine *Debaryomyces hansenii* and *Stenotrophomonas rhizophila* and their potential modes of action. Postharvest Biol. Technol..

[48] Compant S., Duffy B., Nowak J., Clément C., Barka E.A. (2005). Use of plant growth-promoting bacteria for biocontrol of plant diseases: Principles, mechanisms of action and future prospects. Appl. Environ. Microbiol..

[49] Falardeau J., Wise C., Novitsky L., Avis T. (2013). Ecological and mechanistic insights into the direct and indirect antimicrobial properties of *B. subtilis* lipopeptides on plant pathogens. J. Chem. Ecol..

[50] Karthika S., Remya M., Varghese S., Dhanraj N.D., Sali S., Rebello S., Jose S.M., Jisha M.S. (2022). *Bacillus tequilensis* PKDN31 and *Bacillus licheniformis* PKDL10-As double headed swords to combat *Fusarium oxysporum* f. sp. *lycopersici* induced tomato wilt. Microb. Pathog..

[51] Koumoutsi A., Chen X.H., Henne A., Liesegang H., Hitzeroth G., Franke P., Vater J., Borriss R. (2004). Structural and functional characterization of gene clusters directing nonribosomal synthesis of bioactive cyclic lipopeptides in *Bacillus amyloliquefaciens* strain FZB42. J. Bacteriol..

[52] Raaijmakers J.M., De Bruijn I., Nybroe O., Ongena M. (2010). Natural functions of lipopeptides from *Bacillus* and *Pseudomonas*: More than surfactants and antibiotics. FEMS Microbiol. Rev..

[53] Yaraguppi D.A., Bagewadi Z.K., Patil N.R., Mantri N. (2023). Iturin: A promising cyclic lipopeptide with diverse applications. Biomolecules.

[54] Xue J., Liang J., Jiang H., Yin K. (2023). Lipopeptides produced by *Bacillus subtilis* B25 contribute to the biocontrol of gray mold disease. Front. Microbiol..

[55] Tendulkar S., Saikumari Y., Patel V., Raghotama S., Munshi T., Balaram P., Chattoo B. (2007). Isolation, purification and characterization of an antifungal molecule produced by *Bacillus licheniformis* BC98 and its effect on phytopathogen *Magnaporthe grisea*. J. Appl. Microbiol..

[56] Chen X.H., Scholz R., Borriss M., Junge H., Mögel G., Kunz S., Borriss R. (2009). Difficidin and bacilysin produced by plant-associated *Bacillus amyloliquefaciens* are efficient in controlling fire blight disease. J. Biotechnol..

[57] Abubacker M.N., Devi P.K. (2014). In vitro antifungal potentials of bioactive compound oleic acid, 3-(octadecyloxy) propyl ester isolated from *Lepidagathis cristata* Wild. (Acanthaceae) inflorescence. Asian Pac. J. Trop. Med..

[58] Mousa A.A.A., Mahmoud W.H., Elsaied H.E., Elbeltagy A.E. (2025). *Halomonas* sp. for sustainable agriculture: A potential halo bio fertilizer for tomato plants with biocontrol activity against *Fusarium* wilt under saline environments. Sci. Rep..

[59] Shittu A.O., Njinga N.S. (2018). Extraction, purification and determination of phyto-component of fatty acids from *Irvingia gabonensis* seeds. Int. J. Pharmacogn..

[60] Nitbani F.O., Jumina J. (2020). Monoglycerides as an antifungal agent. Apolipoproteins, Triglycerides and Cholesterol.

[61] AH Ibrahim H., S Amer M., O Ahmed H., A Hassan N. (2020). Antimicrobial activity of the sea hare (*Aplysia fasciata*) collected from the Egyptian Mediterranean Sea, Alexandria. Egypt. J. Aquat. Biol. Fish..

[62] Idris F.N., Abd Shukor S.R., Nadzir M.M. (2022). Phytochemicals, antioxidant and antimicrobial activity of *Centella asiatica* extracted by solvent-free microwave extraction. ASEAN Eng. J..

[63] Ahsan T., Chen J., Zhao X., Irfan M., Wu Y. (2017). Extraction and identification of bioactive compounds (eicosane and dibutyl phthalate) produced by *Streptomyces* strain KX852460 for the biological control of *Rhizoctonia solani* AG-3 strain KX852461 to control target spot disease in tobacco leaf. AMB Express.

[64] Bhat M.P., Kumar R.S., Chakraborty B., Nagaraja S.K., Babu K.G., Nayaka S. (2024). Eicosane: An antifungal compound derived from *Streptomyces* sp. KF15 exhibits inhibitory potential against major phytopathogenic fungi of crops. Environ. Res..

[65] Li X.C., Jacob M.R., Khan S.I., Ashfaq M.K., Babu K.S., Agarwal A.K., ElSohly H.N., Manly S.P., Clark A.M. (2008). Potent in vitro antifungal activities of naturally occurring acetylenic acids. Antimicrob. Agents Chemother..

[66] Behera H.T., Mojumdar A., Behera S.S., Das S., Ray L. (2022). Biocontrol of wilt disease of rice seedlings incited by *Fusarium oxysporum* through soil application of *Streptomyces chilikensis* RC1830. Lett. Appl. Microbiol..

