# Supplementary material for: Comparative Insights into Bacterial Endophytome and Antifungal Metabolites in Fusarium Wilt-Resistant and -Susceptible Banana Cultivars Unveil the Resistance Toward Fusarium oxysporum f. sp. cubense
Source: Pathogens. 2026 Aug 19;15(8):864. doi: 10.3390/pathogens15080864 (PMC13516698; doi:10.3390/pathogens15080864)
Supplement: Supplementary file 1 [file pathogens-15-00864-s001.zip › pathogens-4416428-supplementary.pdf]

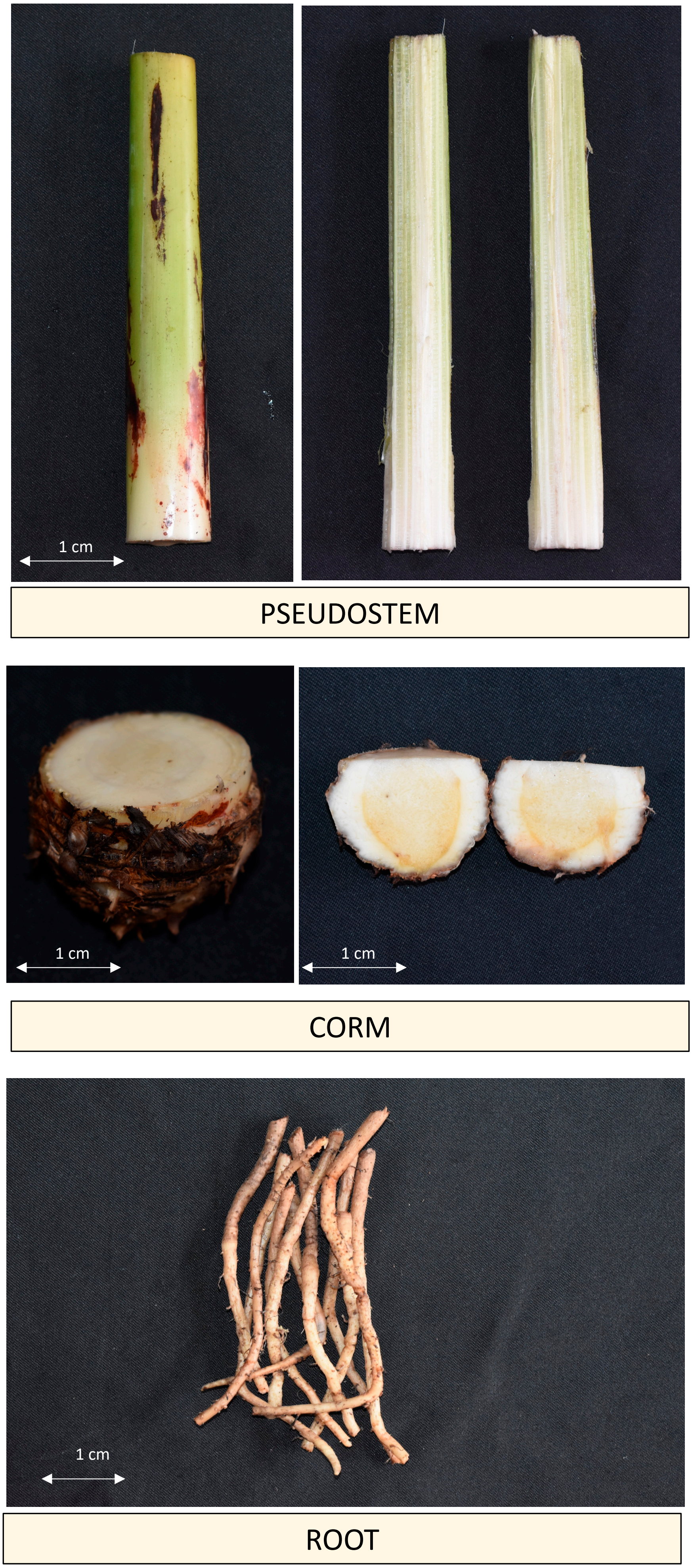

Supplementary figure S1. Pseudostem, corm, and root of banana (*Musa* spp.) used as sources for endophytic bacterial isolation
